# EBV Nuclear Antigen 3C Mediates Regulation of E2F6 to Inhibit E2F1 Transcription and Promote Cell Proliferation

**DOI:** 10.1371/journal.ppat.1005844

**Published:** 2016-08-22

**Authors:** Yonggang Pei, Shuvomoy Banerjee, Zhiguo Sun, Hem Chandra Jha, Abhik Saha, Erle S. Robertson

**Affiliations:** 1 Department of Otorhinolaryngology-Head and Neck Surgery, and the Tumor Virology Program, Abramson Comprehensive Cancer Center, Perelman School of Medicine at the University of Pennsylvania, Philadelphia, Pennsylvania, United States of America; 2 Department of Biological Sciences, Presidency University, Kolkata, India; Tulane Health Sciences Center, UNITED STATES

## Abstract

Epstein–Barr virus (EBV) is considered a ubiquitous herpesvirus with the ability to cause latent infection in humans worldwide. EBV-association is evidently linked to different types of human malignancies, mainly of epithelial and lymphoid origin. Of interest is the EBV nuclear antigen 3C (EBNA3C) which is critical for EBV-mediated immortalization. Recently, EBNA3C was shown to bind the E2F1 transcription regulator. The E2F transcription factors have crucial roles in various cellular functions, including cell cycle, DNA replication, DNA repair, cell mitosis, and cell fate. Specifically, E2F6, one of the unique E2F family members, is known to be a pRb-independent transcription repressor of E2F-target genes. In our current study, we explore the role of EBNA3C in regulating E2F6 activities. We observed that EBNA3C plays an important role in inducing E2F6 expression in LCLs. Our study also shows that EBNA3C physically interacts with E2F6 at its amino and carboxy terminal domains and they form a protein complex in human cells. In addition, EBNA3C stabilizes the E2F6 protein and is co-localized in the nucleus. We also demonstrated that both EBNA3C and E2F6 contribute to reduction in E2F1 transcriptional activity. Moreover, E2F1 forms a protein complex with EBNA3C and E2F6, and EBNA3C competes with E2F1 for E2F6 binding. E2F6 is also recruited by EBNA3C to the E2F1 promoter, which is critical for EBNA3C-mediated cell proliferation. These results demonstrate a critical role for E2F family members in EBV-induced malignancies, and provide new insights for targeting E2F transcription factors in EBV-associated cancers as potential therapeutic intervention strategies.

## Author Summary

EBV is associated with a broad range of human cancers. EBV-encoded nuclear antigen 3C (EBNA3C) is one of the essential latent antigens important for deregulating the functions of numerous host transcription factors which play vital roles in B-cell immortalization. The family of E2F transcription factors are involved in diverse cellular functions. More specifically, E2F6 is one of the E2F family members with a unique property of transcriptional repression. Our current study now demonstrates that EBNA3C can enhance E2F6 repressive functions, and is also responsible for increased E2F6 protein expression in EBV-transformed LCLs. EBNA3C directly interacts with E2F6 at its amino and carboxy terminal domains. Additionally, E2F6 was stabilized by EBNA3C and co-localized in nuclear compartments. Our study also demonstrated that EBNA3C and E2F6 expression resulted in decreased transcriptional activity of E2F1, and that EBNA3C, E2F6 and E2F1 can form a protein complex, and EBNA3C competes with E2F1 for E2F6 binding. The recruitment of E2F6 by EBNA3C was also shown to be important for its related cell proliferation. These results showed a crucial role for EBNA3C-mediated deregulation of E2F6 and its impact on the activities of other E2F family members. Our findings also provide new insights for targeting these E2F transcription factors as potential therapeutic intervention strategies in EBV-associated cancers.

## Introduction

EBV nuclear antigen 3C (EBNA3C) is expressed in latent infection and essential for EBV-mediated B-cell transformation, which plays an intricate role in regulation of viral as well as cellular gene transcription [1,2]. A large proportion of cellular transcription factors are genes encoded by oncogenes and tumor suppressors [3]. Deregulated expression of transcription factors are critical contributors to the oncogenic process. Furthermore, the transcription factors involved in most of oncogenic signaling pathways ultimately control downstream gene expression which leads to transformation, tumorigenesis, tumor progression and metastasis. Under normal physiological conditions, these tumor-associated genes are tightly regulated by upstream transcription modulators, whereas their abnormal expression regulated by the transcription factors result in deregulated expression of multiple tumor-related genes and subsequent oncogenic progression.

Interestingly, EBNA3C similar to well-known master regulators can interact with various cellular transcription regulators, including RBP-Jκ/CBF1, PU.1, Spi-B, Nm23-H1, IRF4/8, CtBP, p300, Prothymosin-α, HDAC1, SUMO1, SUMO3 [4,5]. Recently, we have shown that the IRF4 transcription factor is stabilized by EBNA3C, which leads to degradation of its family member IRF8 through the ubiquitin-proteasome pathway and subsequent inhibition of its tumor suppressive activity [5]. Also, EBNA3C was found to inhibit E2F1-mediated apoptosis through blocking its transcription activity, and prompting its degradation in a ubiquitin-proteasome dependent pathway [6]. E2F transcription factors, which involve activators and repressors of this family members, play critical roles in the regulation of gene expression and diverse cellular functions, such as cell cycle, DNA replication, DNA repair, cell mitosis, and cell fate. The E2F repressor members which includes E2F6 have been shown to inhibit the transcription of E2F-responsive genes through a pRb-independent pathway [7].

Previous studies demonstrated that E2F6 appears to behave as a dominant-negative repressor to inhibit the transcription of E2F-target genes by competing with other E2F family members, and can also function as an active inhibitor [8,9]. E2F6 plays an important role in hypoxia-induced apoptosis by regulating the E2F1/Apaf-1 pathway, and suppresses growth-associated apoptosis through counteracting the pro-apoptotic activity of E2F1 in human hematopoietic progenitor cells [10,11]. Enhanced expression of E2F6 can also alter important cell growth parameters. For example, increased E2F6 expression can delay the exit of cells from S phase [12]. However, a detailed mechanism of E2F6-mediated transcription repression needs further investigation.

In our study, we demonstrated that EBNA3C enhances E2F6 expression in EBNA3C expressing cells and EBV-transformed LCLs. EBNA3C is associated with E2F6 specifically, and also forms a complex by interacting with E2F6 at its amino and carboxy terminal domains. In addition, EBNA3C was shown to be important for E2F6 stabilization and co-localized with E2F6 in the nucleus. Importantly, EBNA3C and E2F6 can both contribute to the reduction of E2F1 transcriptional activity, and compete with E2F1 for E2F6 binding. Therefore, EBNA3C-mediated deregulation of E2F6 and its impact on other E2F family members is an important contributor to EBV-associated malignancies. Our findings also infer that E2F6 or other E2F transcription factors can be targeted as potential therapeutic intervention strategies in EBV-associated cancers.

## Results

### EBV modulates expression of E2F6 and other E2F family members

**Fig 1:**
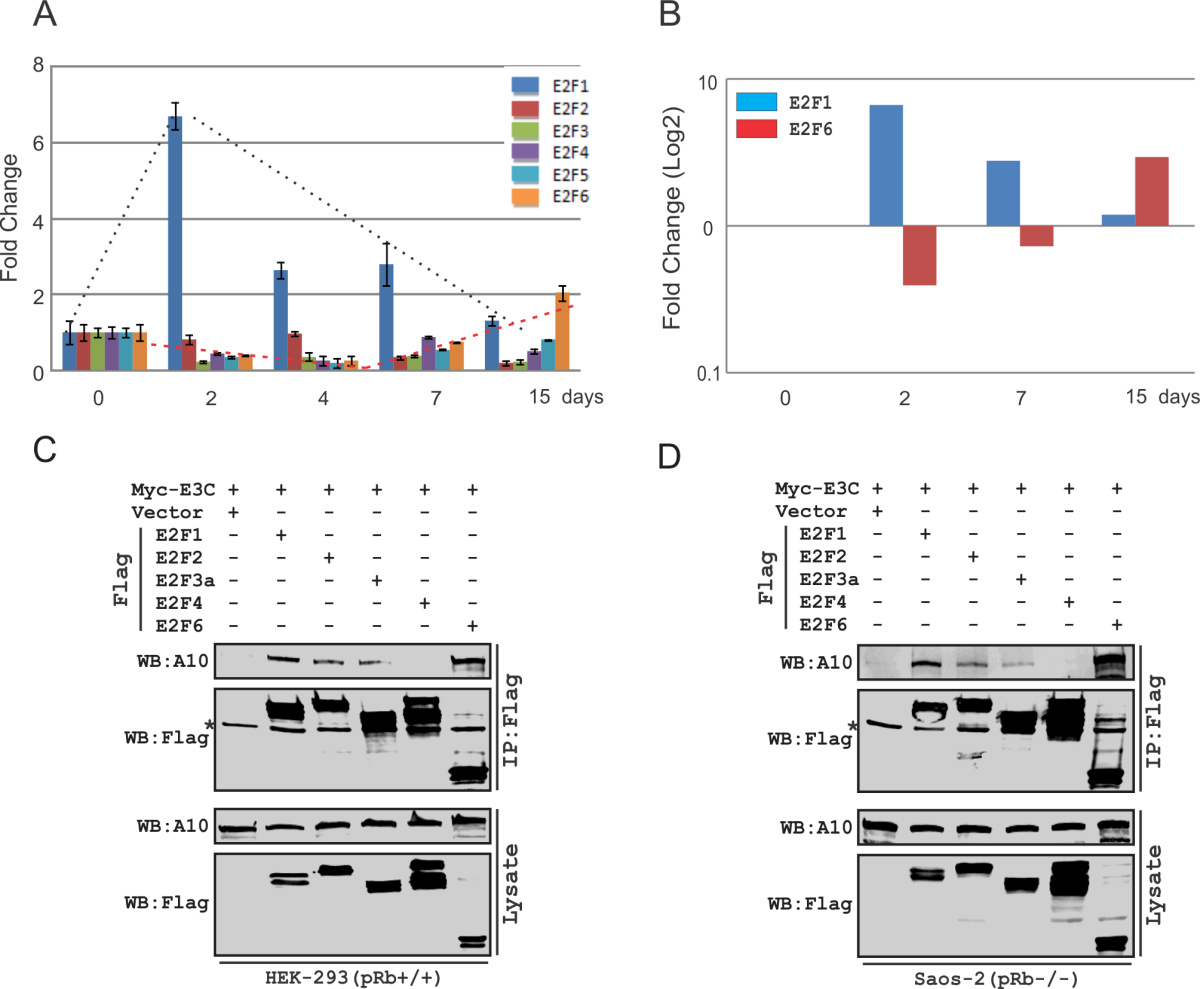
EBV modulates expression of E2F6 and other E2F family members. A) 10 million human peripheral blood mononuclear cells (PBMC) were infected with BAC-GFP-EBV for 4 hours. Cells were harvested at indicated time periods, and total RNA was isolated and reverse transcribed to cDNA according to the manufacturer’s instructions. Quantitative Real-time PCR was performed to detect E2Fs transcription levels. B) Bar graph shows log2 fold changes of E2F1 and E2F6 mRNA expression in detail that were analyzed previously. C-D) 10 million HEK-293 or Saos-2 cells were co-transfected with Myc-EBNA3C and control plasmid or Flag-tagged E2F1, E2F2, E2F3a, E2F4, E2F6 expression plasmids. Transfected cells were harvested and lysed at 36 hours post-transfection. About 5% of total lysates were reserved as the input. After incubating with 1 μg of mouse anti-Flag (M2) antibody, Immunoprecipitations were prepared for detection. Input and immunoprecipitated proteins were identified with 10% SDS-PAGE and analyzed by western blot using specific antibodies.

To investigate the expression levels of E2F family members after infection of primary cells, human peripheral blood mononuclear cells (PBMC) were infected with wild-type BAC-GFP-EBV, and cells were then collected at selected time points (0, 2, 4, 7 and 15 days post-infection). The mRNAs were isolated and detected by subsequent Real-time PCR. The results demonstrated that the E2F family members have a unique pattern of expression after EBV infection (Fig 1A). Most of E2F family members were down-regulated except E2F1. Interestingly, the opposite trend was seen for expression of E2F1 and E2F6, which suggests that E2F1 and E2F6 may have opposite roles during EBV infection (Fig 1B).

Our previous studies demonstrated that EBNA3C can act as a modulator for different transcription factors [13] and has the ability to interact with different members of the E2F transcription factor family [5]. To examine the comparative binding ability of E2F6 and other E2F family transcription factors with EBNA3C, HEK-293 and Saos-2 cells were transfected with E2F1, E2F2, E2F3a, E2F4 and E2F6 expression plasmids with EBNA3C expression construct. The results indicated that E2F1, E2F2, E2F3a, and especially E2F6 can strongly bind with EBNA3C (Fig 1C and 1D, S1A Fig).

### EBNA3C is an important contributor to the enhanced expression of E2F6

In the context of viral infection, abnormal expression of E2F6 was observed as an independent contributor to nasopharyngeal carcinogenesis [14]. To exploit the role of EBNA3C in modulating E2F6 expression, E2F6 mRNA levels were detected in BJAB cells with wild-type or ΔEBNA3C BAC-GFP-EBV infection. The results showed that E2F6 could be up-regulated at 1 day post-infection (dpi) in both wild-type and mutant virus infection. However, E2F6 expression was stable in wild-type EBV infected cells and increased to higher levels at 7 dpi, while its expression was not up-regulated in ΔEBNA3C BAC-GFP-EBV infected cells (S2A Fig). Meanwhile, the highest level of EBNA3C expression was also observed at 7 dpi (S2B Fig). We believe that this strongly supports our hypothesis that E2F6 expression is specifically up-regulated by EBNA3C.

**Fig 2:**
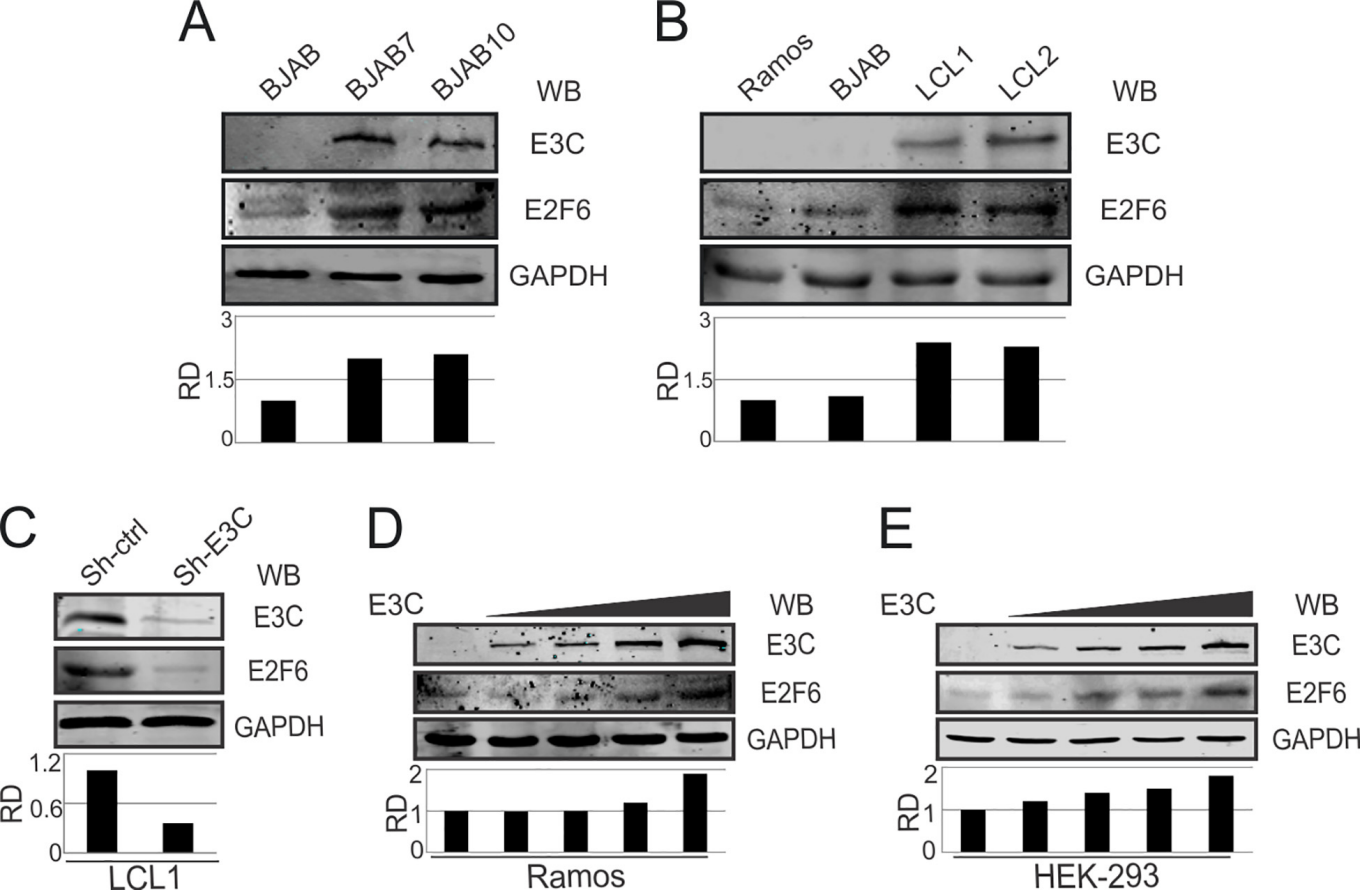
EBNA3C is an important contributor to the enhanced expression of E2F6. A-C) 10 million EBV-negative BJAB, Ramos, EBNA3C stable expressing BJAB7, BJAB10, EBV-transformed LCL1, LCL2, and sh-ctrl (stable control knockdown), sh-EBNA3C (stable EBNA3C knockdown) LCL1 cells were harvested and lysed. Western blot analysis of E2F6 protein expression levels was shown with indicated antibodies. D-E) 10 million Ramos or HEK-293 cells were co-transfected with increasing doses of EBNA3C expressing constructs (0, 5, 10, 15, 20 μg) and E2F6, EBNA3C, GAPDH proteins were detected with western blot analysis. GAPDH was used as an internal loading control.

To further prove that E2F6 up-regulation is indeed mediated by EBNA3C, E2F6 protein levels were determined by western blot using E2F6-specific antibody in EBV-negative BJAB, and EBNA3C stable expressing BJAB7, BJAB10 cells. Our results clearly showed enhanced E2F6 expression in BJAB7, BJAB10 cells compared to BJAB cells (Fig 2A). We extended our studies in EBV-transformed LCL1, LCL2 cells and also observed higher E2F6 expression in those cells in comparison with EBV-negative Ramos, BJAB cells (Fig 2B). In addition, the up-regulation of E2F6 expression was also observed from the naturally isolated Burkitt's lymphoma cells Mutu Type III (latency III) when compared with Mutu Type I (latency I) (S1B Fig). Interestingly, stable knockdown of EBNA3C in EBV-transformed LCL1 cells resulted in a substantial reduction in E2F6 protein levels (Fig 2C). Furthermore, increasing amounts of an EBNA3C expression plasmid were transfected into EBV-negative Ramos and HEK-293 cells. Subsequent western blot analysis revealed that increased EBNA3C expression results in a gradual increase of E2F6 protein levels (Fig 2D and 2E), suggesting a specific role of EBNA3C in upregulating E2F6 protein expression.

### E2F6 binds to both amino and carboxy terminal domains of EBNA3C

**Fig 3:**
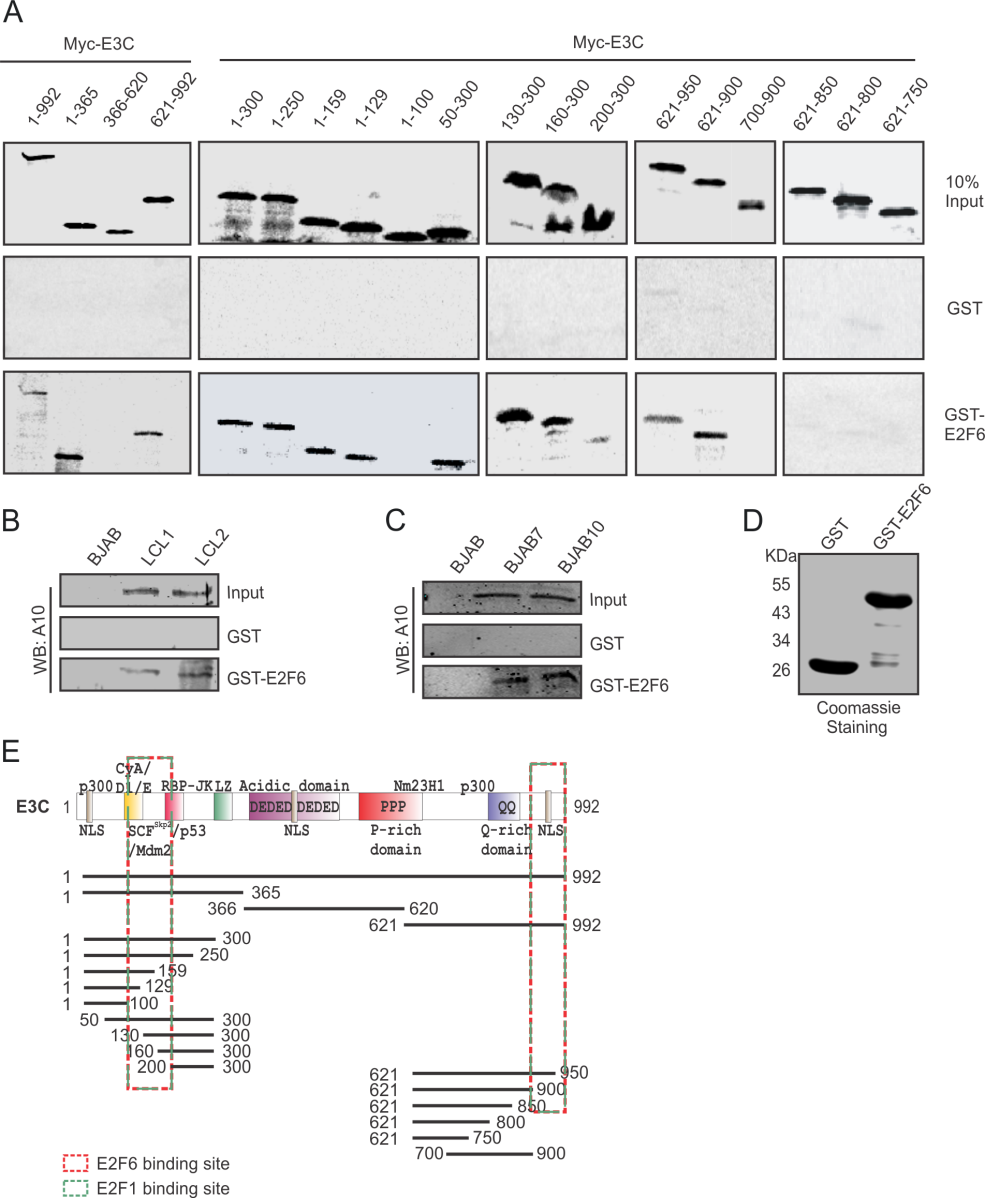
E2F6 binds to both amino and carboxy terminal domains of EBNA3C. A) Myc-tagged EBNA3C full length and different truncated mutants were translated *in vitro* using a T7-TNT kit. The S^35^-radiolabeled *in vitro*-translated proteins were pre-cleared with GST-beads, then incubated with GST control or GST-E2F6 protein and GST beads. The mixture were resolved by 10% SDS-PAGE, exposed to phosphorImager plates and scanned with Typhoon Scanner. B-C) Cell lysates form 50 million different B-cells (BJAB, BJAB7, BJAB10, LCL1, LCL2) were collected and incubated with either GST control or GST-E2F6 protein and GST beads. The mixture samples were identified by western blot analysis using specific mouse antibody for EBNA3C (A10). D) Purified GST control and GST-E2F6 proteins were separated by 10% SDS-PAGE and stained with Coomassie Blue. E) The schematic diagram shows various interactive domains of EBNA3C with E2F6.

In order to map the binding domain of EBNA3C with E2F6, we performed GST pull-down assays using full length EBNA3C and its different truncated mutants. The indicated proteins were produced by *in vitro* transcription/translation assays and co-incubated with purified GST-E2F6 protein generated from bacteria. The interacting domains were identified by western blot analysis. Our results demonstrated that the N-terminal domain (residues 100–200) and C-terminal domain (residues 900–992) of EBNA3C can directly interact with E2F6 (Fig 3A and 3E). Additionally, we also performed GST pull-down assays in BJAB, BJAB7, BJB10, LCL1, LCL2 cells and observed strong association of E2F6 with EBNA3C (Fig 3B, 3C and 3D). These results further corroborates our above results that EBNA3C physically associates in a complex with E2F6 *in vitro*.

### EBNA3C forms a protein complex with E2F6 in EBV-transformed B-cells

**Fig 4:**
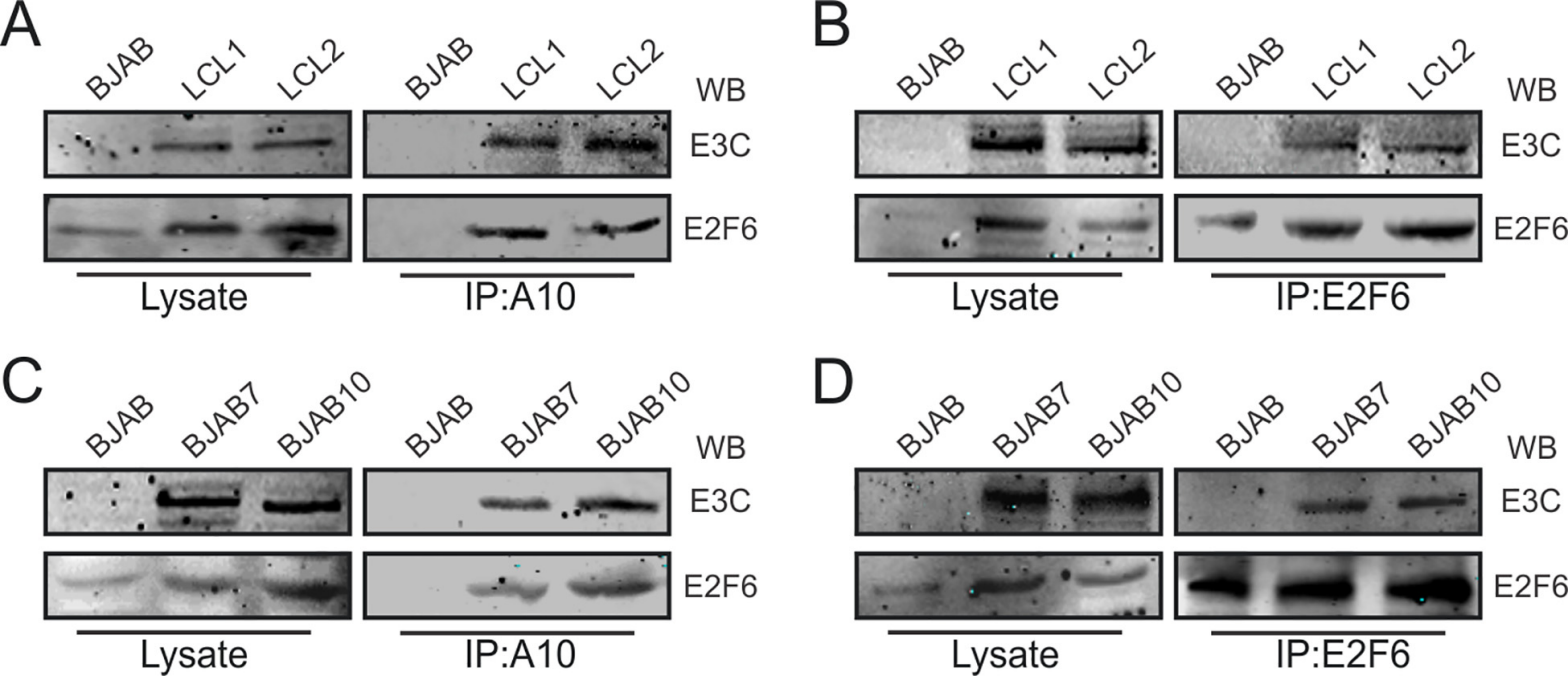
EBNA3C forms a complex with E2F6 in EBV-transformed B-cells. A-D) Cell lysates from different B-cell lines (BJAB, BJAB7, BJAB10, LCL1, and LCL2) were immunoprecipitated with E2F6 or EBNA3C specific antibody (A10). Then immunoprecipitated samples were analyzed with western blot and endogenous EBNA3C, E2F6 proteins were detected by the indicated antibodies.

To determine the endogenous complexes which would demonstrate association of EBNA3C with E2F6 in the B-cell background, we performed co-immunoprecipitation assays in different B-cell lines, including BJAB, BJAB7, BJAB10, LCL1 and LCL2 cells. These results clearly showed that EBNA3C interacts specifically with E2F6 in EBNA3C stably expressing cells as well as EBV-transformed B-cells (Fig 4). Using specific EBNA3C antibodies, we showed that by co-immunoprecipitation assays E2F6 was brought down in this complex (Fig 4A). The reverse co-immunoprecipitation assay showed that with E2F6 specific antibodies EBNA3C was precipitated with the complex (Fig 4B). Similarly, co-immunoprecipitation experiments performed in EBNA3C stable expressing B-cell lines demonstrated a protein complex of EBNA3C and E2F6 (Fig 4C and 4D).

### EBNA3C co-localizes with E2F6 in nuclear compartments in transformed LCLs

**Fig 5:**
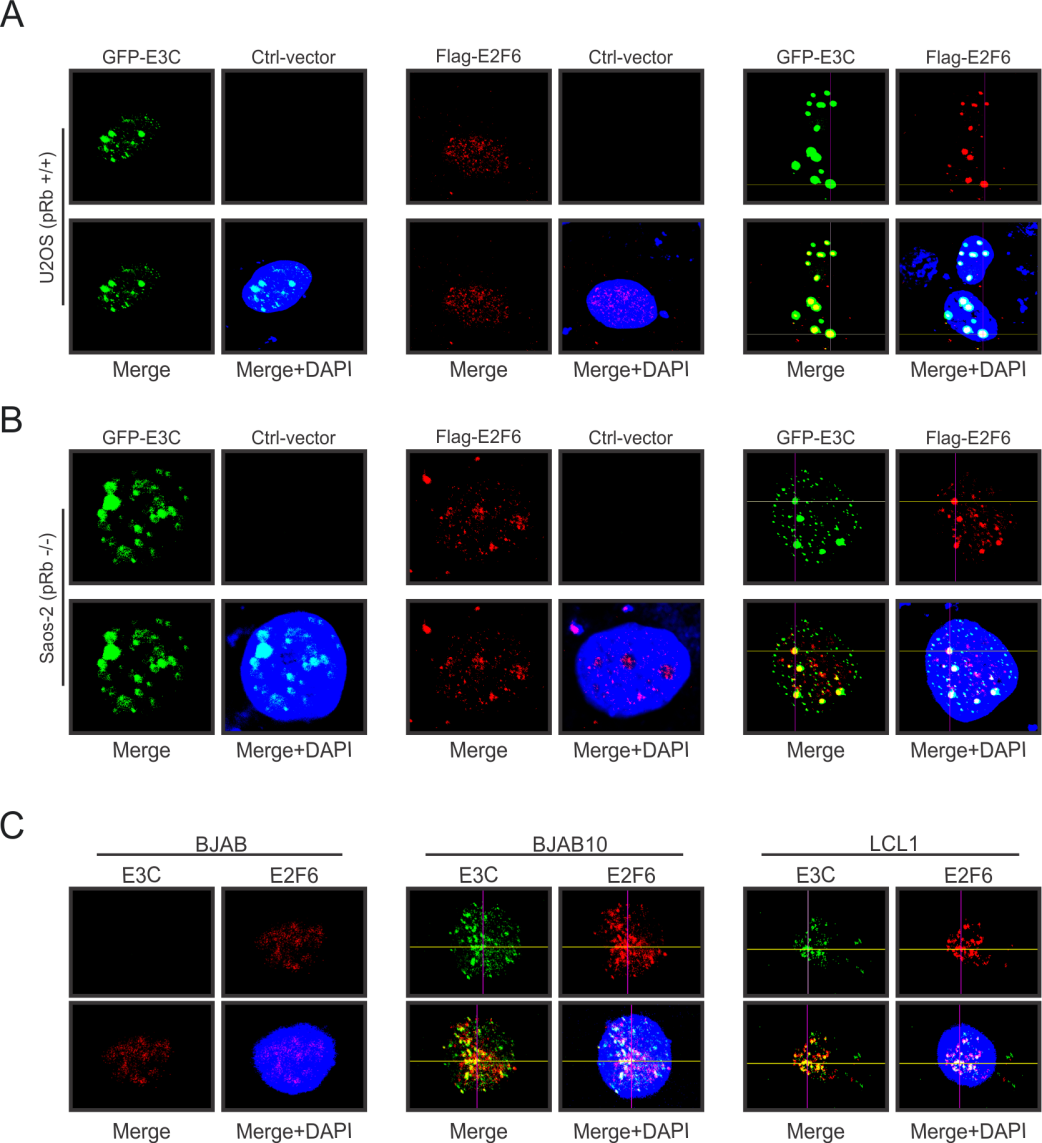
EBNA3C co-localizes with E2F6 in nuclear compartments in transformed LCLs. A-B) 0.3 million U2OS or Saos-2 cells were seeded on coverslips and transiently transfected with control plasmid, GFP-EBNA3C or Flag-E2F6 expression constructs using Polyplus jetPRIME. Exogenous E2F6 protein was incubated with mouse anti-Flag (M2) antibody and Alexa Fluor 594-conjugated secondary antibody, while GFP-tagged EBNA3C was detected by GFP-fluorescence directly. C) BJAB, BJAB10, LCL1 cells were semi-air-dried on slides. Endogenous EBNA3C and E2F6 proteins were detected with their specific primary antibodies, followed by the corresponding secondary antibodies. And the nuclei was stained with DAPI. The images were captured using Olympus Fluoview confocal microscope and analyzed with FLUOVIEW software.

To examine whether the association of EBNA3C and E2F6 takes place in the same cellular compartments, we initially performed immunofluorescence experiments by transfecting GFP-EBNA3C and Flag-E2F6 plasmids into U2OS or Saos-2 cells. Our studies showed that EBNA3C signals were punctate foci with exclusion of nuclei as expected, and E2F6 signal exhibited an intense staining pattern of prominent foci in the nucleus. Importantly, there were a number of places where co-localization staining of EBNA3C with E2F6 was evident (Fig 5A and 5B). To further validate the interaction between these two proteins under more relevant physiological conditions, BJAB, BJAB10 and LCL1 cells were used in the following immunofluorescence assays. Our results clearly demonstrated that endogenous EBNA3C and E2F6 strongly co-localized in BJAB10 and LCL1 cells compared with BJAB cells, supporting the above evidence that they formed a molecular complex in nuclear compartments in EBNA3C stable expressing as well as EBV-transformed B-cells (Fig 5C). Additionally, the co-localization assay was extended using different mutants of EBNA3C and the results demonstrated that N-terminal or C-terminal of EBNA3C could co-localize with E2F6 in nucleus, while the middle domain (residues 366–620) of EBNA3C showed negligible co-localization (S4 Fig).

### E2F6 is stabilized by EBNA3C

**Fig 6:**
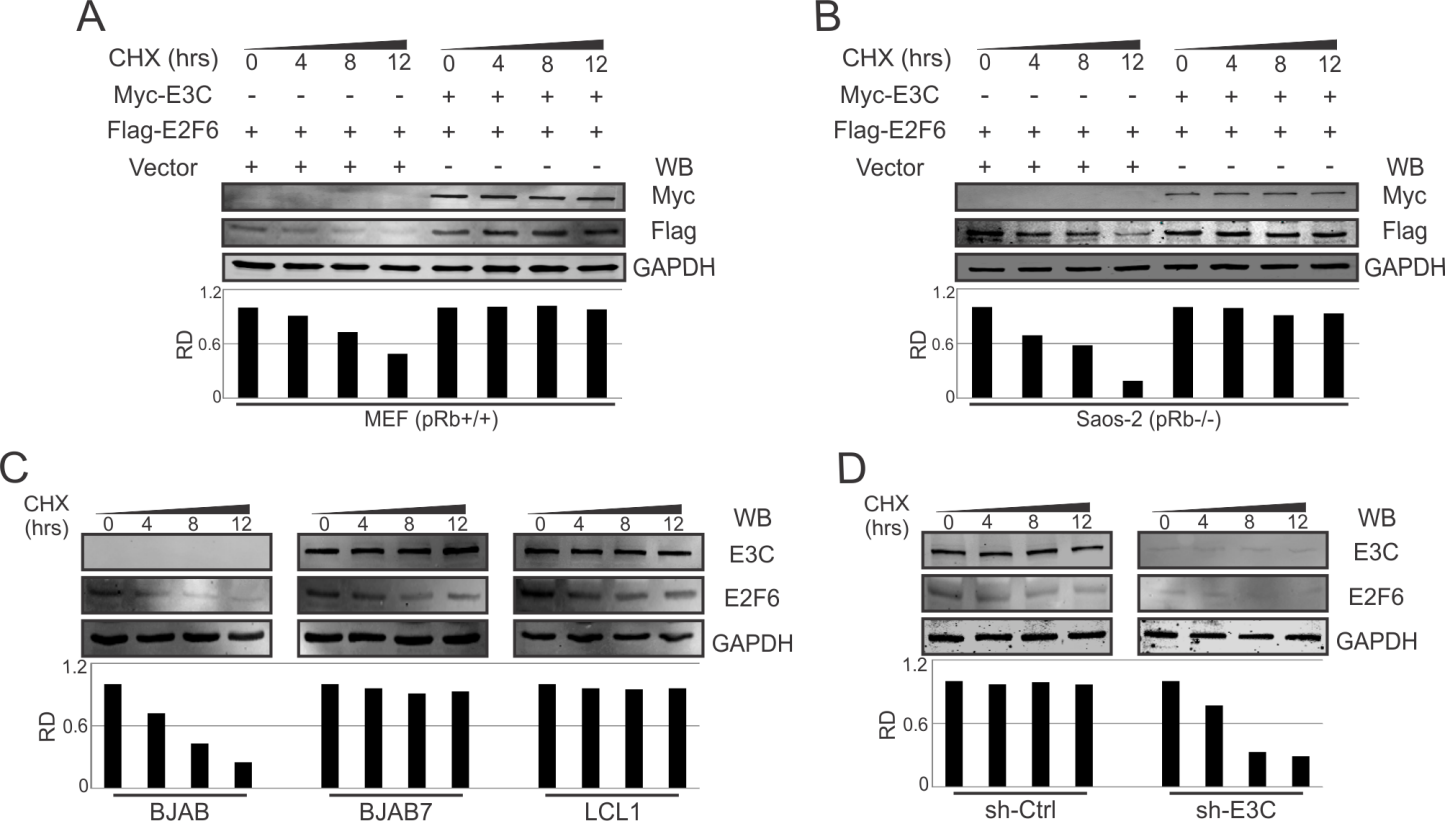
E2F6 is stabilized by EBNA3C. A-B) 10 million MEF or Saos-2 cells were co-transfected with control plasmids, Myc-E3C, and Flag-EBNA3C expression plasmids. At 36 hours post-transfection, cells were treated with 40 μg/ml cycloheximide (CHX) for 0, 4, 8, 12 hours, and then lysed and analyzed by immunoblotting with specific antibodies. C-D) Different B-cells (BJAB, BJAB7, LCL1, and sh-ctrl, sh-EBNA3C LCL1) were obtained after treating with 40 μg/ml cycloheximide (CHX) for indicated time periods, and analyzed by western blot with specific antibodies. GAPDH was used as an internal loading control.

Our previous results indicated that E2F6 protein levels were significantly enhanced by EBNA3C, further suggesting an important role of EBNA3C for the maintenance of E2F6 protein expression. To further validate the observations that EBNA3C-induced E2F6 protein expression levels were due to enhanced E2F6 protein stability, we executed protein stability assays by expressing E2F6 with or without EBNA3C in MEF and Saos-2 cells. These studies showed that E2F6 was stabilized on expression of EBNA3C (Fig 6A and 6B). We further extended this experiment by treating BJAB, BJAB7, LCL1, and sh-ctrl (stable control knockdown), sh-EBNA3C (stable EBNA3C knockdown) LCL1 cells with cycloheximide (CHX). The results showed that the stability of E2F6 was significantly enhanced in the presence of EBNA3C in comparison with a loss of greater than 75% of the E2F6 signal by 12 hours post-cycloheximide treatment in EBNA3C-negative cells (Fig 6C and 6D, S1C Fig).

### EBNA3C and E2F6 can repress the transcriptional activity of E2F1

**Fig 7:**
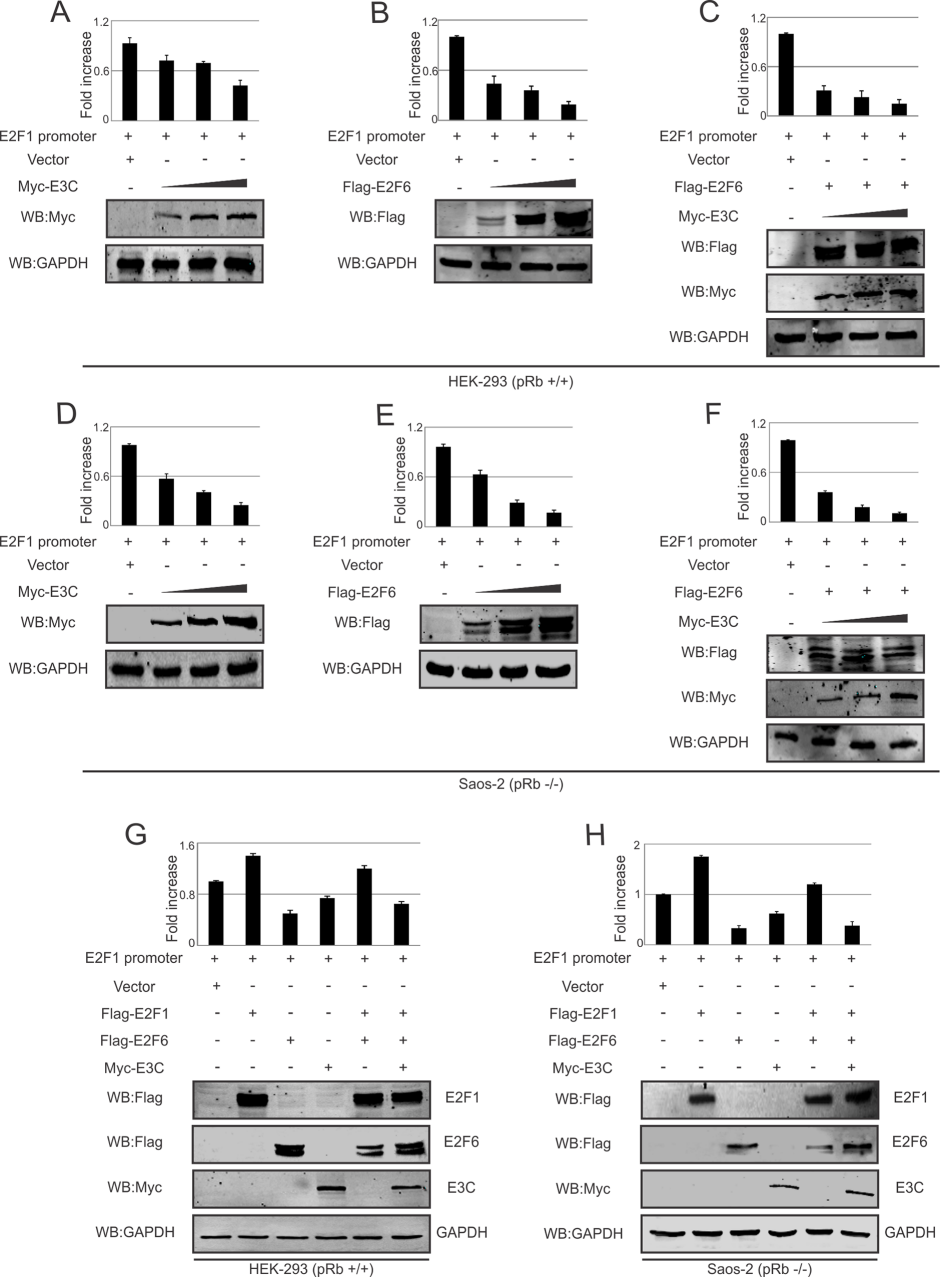
EBNA3C and E2F6 can repress the transcriptional activity of E2F1. A-F) 10 million HEK-293 or Saos-2 cells were transfected with pGL2 control vector, full-length E2F1 promoter construct, Flag-E2F6, either increasing doses of EBNA3C or E2F6 expression plasmids. Thirty-six hours post-transfection, cells were harvested and carried out for luciferase reporter assays. Expression of transfected constructs were detected by western blot. G-H) 10 million HEK-293 or Saos-2 cells were transfected with pGL2 control vector, full-length E2F1 promoter construct, Flag-E2F1, Flag-E2F6, and EBNA3C expression plasmids. Luciferase reporter assays were carried out at 36 hours post-transfection as above. Expression levels of transfected constructs were also examined by western blot.

Previous studies suggested that E2F6 can act as a repressor by inhibiting the transcription of known E2F-responsive genes [9]. To determine whether EBNA3C can play an important role in regulating E2F1 promoter activity involving E2F6, we performed promoter luciferase assays by expressing increasing amounts of E2F6, EBNA3C or co-expressing E2F6 with increasing amounts of EBNA3C in the presence of wild-type E2F1 promoter in HEK-293 and Saos-2 cells. These results showed that E2F6 and EBNA3C reduced E2F1 transcriptional activity in a dose-dependent manner in these two cell lines (Fig 7A–7F). Both E2F6 and EBNA3C were effective at independently suppressing the promoter activity. However, when both were added a more dramatic reduction was seen compared to each independent activity (Fig 7, compare A/B, D/E with C and F, respectively). The inhibition of EBNA3C-mediated E2F1 promoter activity was reversed by down-regulating E2F6 expression with sh-E2F6 (S3 Fig). Interestingly, we also showed that E2F1 was able to increase its own promoter activity but again showed a reduction in promoter activity when E2F6 and EBNA3C were co-expressed (Fig 7G and 7H).

### EBNA3C competes with E2F1 for E2F6 binding

**Fig 8:**
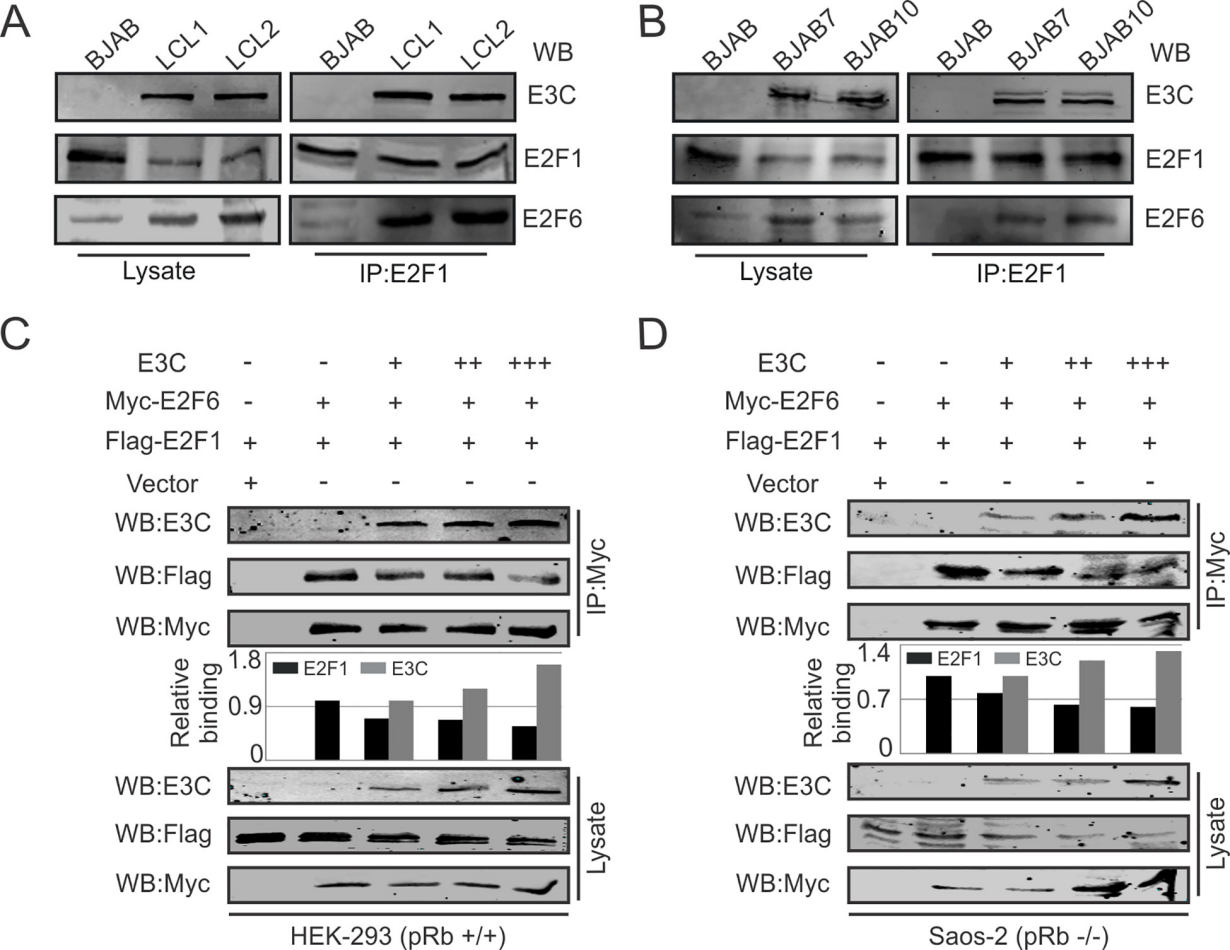
EBNA3C competes with E2F1 for E2F6 binding. A-B) Cell lysates from indicated B-cells (BJAB, BJAB7, BJAB10, LCL1, and LCL2) were immunoprecipitated with E2F1 specific antibody. Then immunoprecipitations were analyzed by western blot to identify expressions of endogenous EBNA3C, E2F6 proteins. C-D) 10 million HEK-293 or Saos-2 cells were electroporated with indicated combinations of Myc-E2F6, Flag-E2F1, EBNA3C expression constructs. The cell lysates were immunoprecipitated using anti-Myc antibody and the immunoprecipitations were examined with western blot.

Our previous binding assays above demonstrated that E2F6 interacts with EBNA3C in the same region as E2F1. Therefore, it is possible that E2F1 can form a molecular complex with E2F6 and EBNA3C. To validate this hypothesis, co-immunoprecipitation experiments were performed in B-cells. Our results showed that E2F1 can form a protein complex with E2F6 and EBNA3C in EBV-transformed, EBNA3C stable expressing cells (Fig 8A and 8B). Furthermore, competitive protein-binding assays were performed in HEK-293 and Saos-2 cells by transfecting increasing amounts of EBNA3C expression plasmids with Myc-E2F6 and Flag-E2F1. The proteins of interest were examined by co-immunoprecipitation using anti-Myc antibody. Our results showed that increasing amounts of EBNA3C can lead to a dose-responsive reduction of binding between E2F1 with E2F6 (Fig 8C and 8D).

### EBNA3C promotes E2F6 binding to the E2F1 promoter

**Fig 9:**
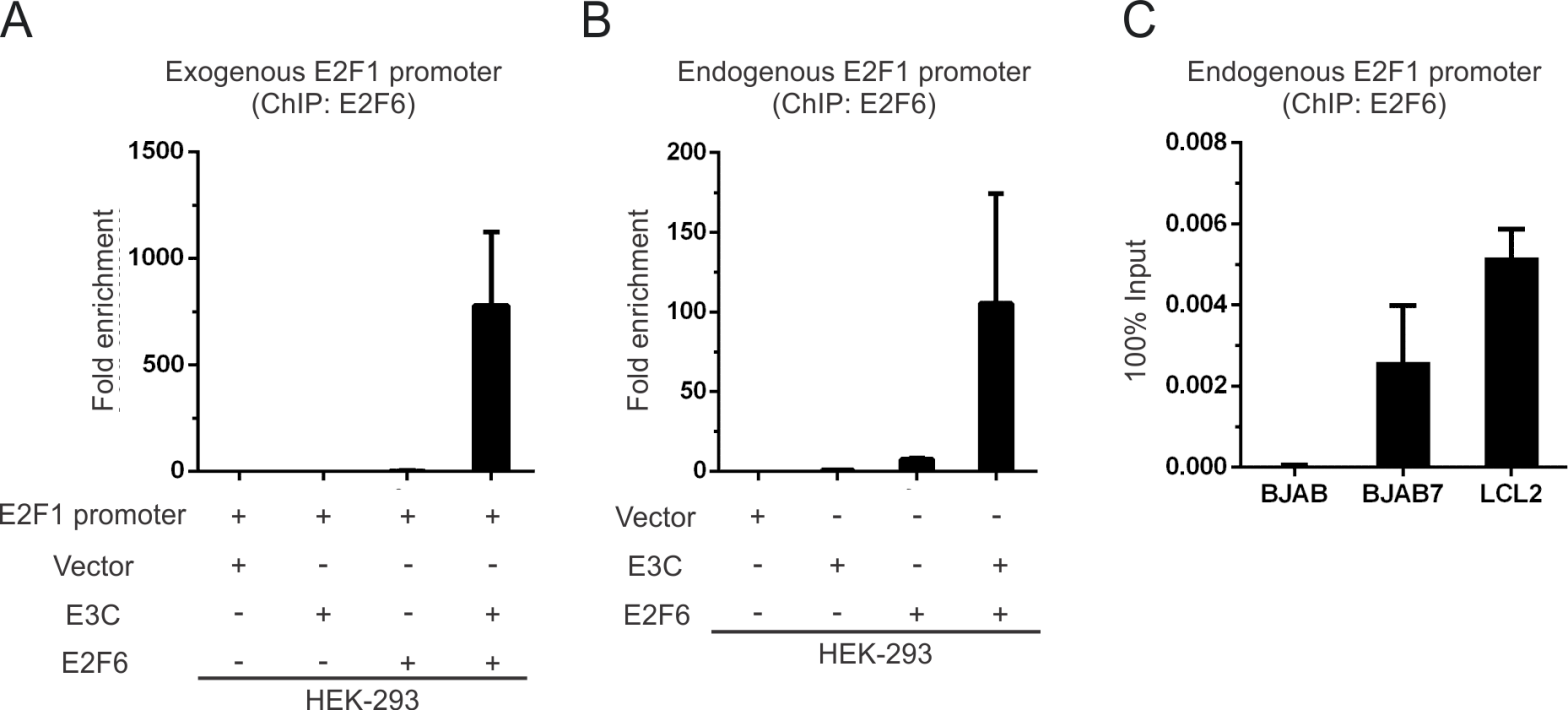
EBNA3C promotes E2F6 binding to E2F1 promoter. A-B) HEK-293 cells were co-transfected with appropriate combinations of Myc-EBNA3C, Flag-E2F6, and pGL2-E2F1 promoter reporter plasmid or pGL2 control plasmid. Then the ChIP assay was performed with E2F6 antibody and recovered DNA was quantitated by Real-time PCR using primers specific for transfected E2F1 promoter (A) or endogenous E2F1 promoter (B). NC, negative control. C) BJAB, BJAB7, LCL2 cells were harvested directly and performed ChIP assay as previously mentioned. The recovered DNA was detected with primers specific for endogenous E2F1 promoter. Shown are the results from independent experiments. Error bars indicate standard deviations.

It has been shown earlier that E2F6 regulated E2F1 expression through the trans-repression of the E2F1 promoter [10]. To explore the effect of EBNA3C on E2F6 recruitment to the E2F1 promoter, a ChIP assay was executed using E2F6 antibody in different cell lines (HEK-293 and BJAB, BJAB7, LCL2 cells). The transfected or endogenous E2F1 promoter DNA were detected by real-time PCR in HEK-293 cells. Our results demonstrated that both the transfected and endogenous E2F1 promoter DNA were significantly immunoprecipitated when E2F6 and EBNA3C were co-expressed compared with the control groups (Fig 9A and 9B). This suggests that accumulation of E2F6 at the E2F1 promoter is greatly enhanced by EBNA3C. Moreover, similar results were also observed in BJAB7 and LCL2 cells (Fig 9C). These results showed that the presence of EBNA3C obviously increased the binding of E2F6 on E2F1 promoter.

### E2F6 is critical for EBNA3C-mediated cell proliferation

**Fig 10:**
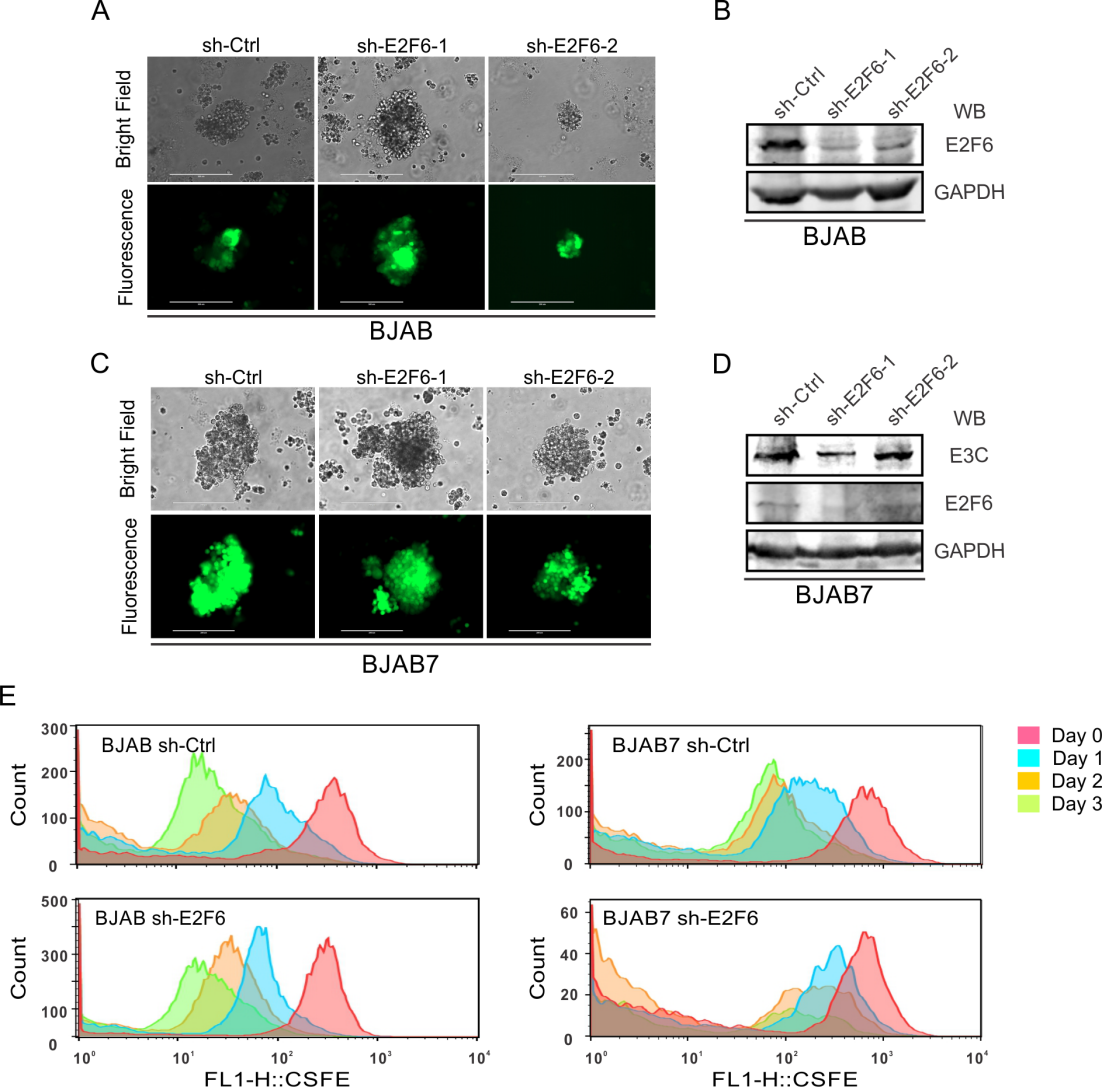
E2F6 is critical for EBNA3C-mediated cell proliferation. A-B) 1 million BJAB cells were infected with the indicated lentivirus in combination 20 μg/ml polybrene. After 72 hours incubation, puromycin antibiotic was added as a 0.5 μg/ml concentration for selection. The selected cells were observed the GFP immunofluorescence (A) and the target protein expressions were detected by western blot (B). C-D) 1 million BJAB7 cells were infected and selected for 2 weeks using 0.5 μg/ml puromycin. GFP immunofluorescence (C) was checked and the target proteins were also analyzed by western blot (D). E) 3 million BJAB or BJAB7 stable cell lines were treated with 5μM CFSE solution for 10 minutes at room temperature. Then cells were washed, cultured, and harvested at indicated time points (0, 24, 48, and 72h). Flow cytometry was used to analyze CFSE-labeled cells.

Our studies above showed that EBNA3C promotes E2F6-mediated E2F1 inhibition. This then leads to cell proliferation. To further explore whether E2F6 was a key factor in this pathway, stable knockdown of E2F6 in BJAB and BJAB7 cells were generated by lentivirus transduction and selection (Fig 10A and 10C), and E2F6 expression levels were monitored by western blot analysis (Fig 10B and 10D). The following CFSE assay was designed to detect cell proliferation in stable E2F6 knockdown B-cell lines. The results demonstrated that E2F6 knockdown in BJAB cells did not affect cell proliferation. However, in the corresponding BJAB7 cells, which stably expressed EBNA3C, cellular proliferation was significantly inhibited (Fig 10E). To further assess these results in context of EBV, E2F6 knock-down LCL1 stable cell lines were generated, and the cell proliferation was also investigated (S5 Fig). Compared with control cells, E2F6 knock-down indeed inhibits the ability of cellular proliferation in LCLs. This suggested that E2F6 was a critical factor for EBNA3C-mediated cell proliferation, and that E2F6 was recruited by EBNA3C as a major contributor to increased cell proliferation in EBV-transformed LCLs.

## Discussion

E2Fs are a designated large family of transcription factors having one or more conserved DNA binding domains. They bind with the target promoters to regulate their expressions [15]. E2Fs can cooperate to generate a coordinated network of carefully regulated cell cycle and cell proliferation events during cancer development. The E2F family of transcription factors can be categorized into several subgroups based on their potential functional activities. E2F1–E2F3 can be regarded as activating E2Fs [16,17]. They are required for transactivation of the target genes which are involved in transition through the G1/S phase of the cell-cycle, as well as proper cell-cycle progression [16,18,19]. E2F4 and E2F5 are considered to have predominantly repressive activities and bind the pRb protein family members [20]. E2F6, E2F7 and E2F8, are considered to be transcription repressors and regulate downstream gene expression through an Rb-independent mechanism [21,22]. Although E2F family members have been studied for many years, we still know little about how they collaborate to control cellular proliferation or tumorigenesis (S6 Fig). A recent study showed that the dynamic interactions of NF-κB and E2F1/E2F4 can control cellular proliferation [23].

The well-known tumor suppressor Rb directly interacts with the E2F transcription factors and can be recruited to E2F-responsive promoters to control diverse signaling pathways and lead to aberrant cell proliferation or human malignances [24,25]. Studies explaining the Rb/E2F pathway have revealed that it is critical for controlling cell proliferation, and plays central roles in cancer development [26]. Our previous study showed that E2F1 could also be down-regulated by EBV latent protein EBNA3C in Rb-independent pathway [27], but it is still unknown whether this regulation could be mediated by other E2F family members. Our current studies demonstrated that EBNA3C-mediated E2F6/E2F1 interaction in the context of EBV infection, and further provides a deeper understanding of the dynamic regulation of E2Fs in EBV-induced malignancies.

E2F6 represents a third subclass of the E2F family which is likely to show distinct biological characteristics when compared to the other E2Fs. Previous studies suggested that E2F6 appears to function as a dominant negative inhibitor of known E2F-responsive genes through competition with other E2F family members, and also act as an active repressor [8,9]. E2F6 has important roles in regulating hypoxia-induced apoptosis via its modulation of E2F1/Apaf-1 pathway, and repressing growth-associated apoptosis by counteracting the pro-apoptotic activity of E2F1 in human hematopoietic progenitor cells [10,11]. In the context of viral infection, abnormal expression of E2F6 was found as an important contributor to nasopharyngeal carcinogenesis [14].

In the current study, we showed that EBNA3C plays an important role in induction of E2F6 expression in LCLs. Our study also demonstrated the physical association between EBNA3C and E2F6, and that EBNA3C interacts with E2F6 through its amino and carboxy-terminal domains. Our endogenous co-immunoprecipitation assays clearly showed that EBNA3C and E2F6 can specifically associate in a protein complex with each other in B-cells. Recent studies suggested that EBNA3C expression can regulate the stabilization of various oncoproteins, transcription factors and cellular kinases [5,28,29]. We performed stability assays for E2F6 to determine whether EBNA3C has a direct role in stabilizing E2F6 and observed in presence EBNA3C, the E2F6 protein was substantially stabilized. The mechanism of EBNA3C-induced E2F6 stability is likely to be through the control of the ubiquitin proteasome degradation pathway. However, the specific E3 ligase is yet to be identified. Our study also demonstrated that EBNA3C and E2F6 strongly co-localizes in nucleus.

**Fig 11:**
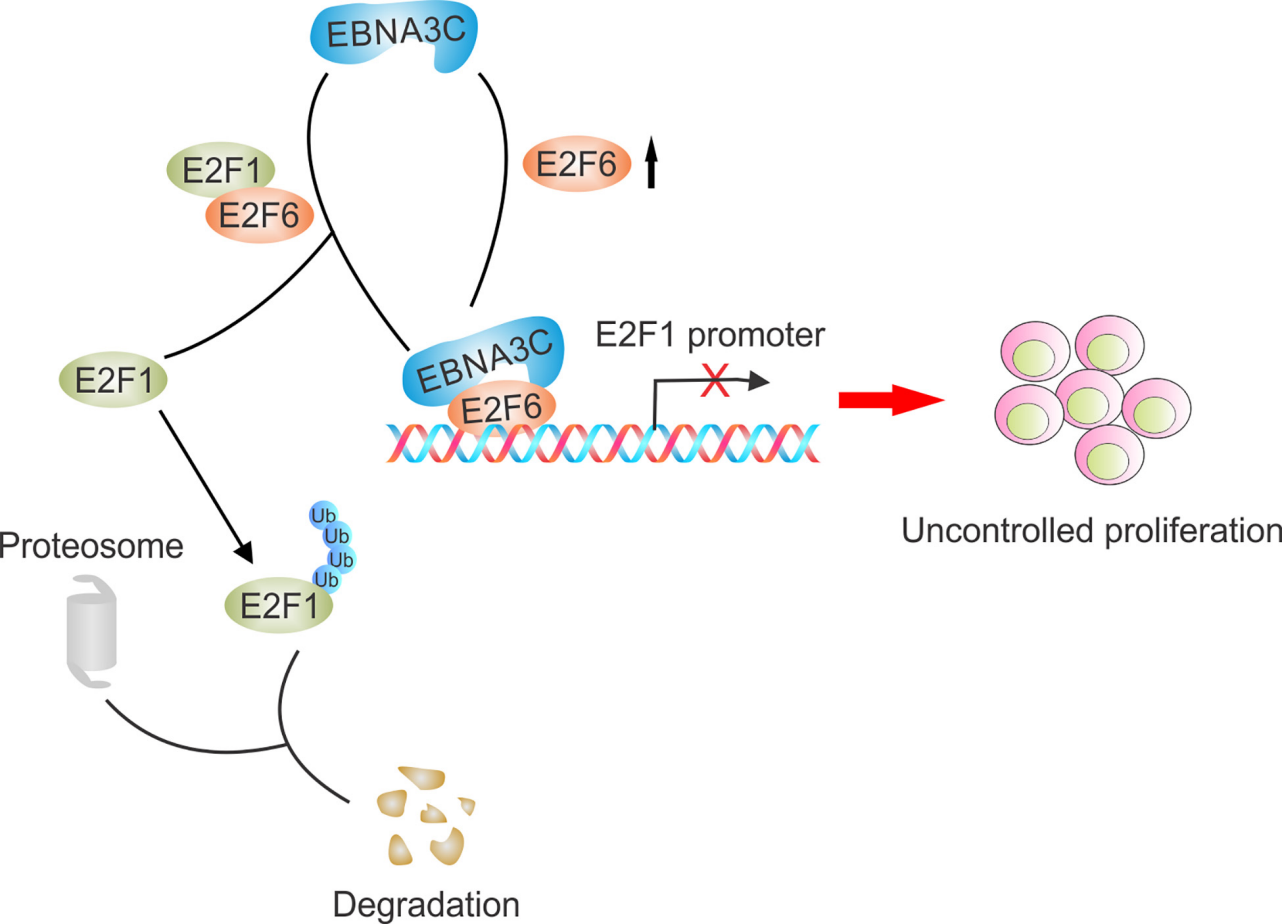
A schematic that illustrates the role of E2F6 in EBNA3C-mediated E2F1 regulation. EBNA3C interacts with E2F6 specifically and enhances the stability of E2F6. EBNA3C can also compete with E2F1 for E2F6 binding. E2F6 recruited together with EBNA3C binds E2F1 promoter and inhibits its activity, which contributes to B-cell proliferation by reducing the expression of E2F1. This mechanism describes that the contribution of E2F6 in EBNA3C-related oncogenic activity important for EBV-transformed B-cell proliferation.

Earlier reports suggested the role of EBV latent antigens in regulation of E2Fs, for example, the EBV protein BRLF1 activates S phase entry through inducing E2F1 expression [30]. Other reports stated that E2Fs are involved in LMP1 mediated downregulation of p27KIP1 transcriptional activity [31]. Nuclear export of E2F4/E2F5 by LMP1 blocked p16INK4a–Rb pathway and regulated cell proliferation [32]. Previously our lab demonstrated that E2F1 mediated apoptosis induced by the DNA damage response was blocked by EBNA3C in lymphoblastoid cells [6]. We now clearly demonstrate that EBNA3C and E2F6 are both responsible for reduction of E2F1 transcriptional activity. Moreover, E2F1 forms a protein complex with EBNA3C and E2F6, and EBNA3C competes with E2F1 for E2F6 binding. E2F6 is also recruited by EBNA3C to bind the E2F1 promoter, and is critical for EBNA3C-mediated cell proliferation (Fig 11). Therefore, in the context of EBV infection, EBNA3C-mediated E2F6/E2F1 regulation may offer novel insights to further understand the important role of EBV latent antigens in E2Fs-related cellular functions or cancer development.

Recently, E2F6 was identified as a component of mammalian Bmi1-containing polycomb complex [33]. The polycomb multi-protein complexes maintain epigenetic regulation of cell type specific gene expression patterns which are critical for cellular identity via histone modification. Dysregulation of these activities promotes oncogenesis by causing inappropriate expression of cell identity, differentiation and proliferation related genes. Interestingly, endogenous E2F6 was found associated with other polycomb group proteins, including RYBP, Ring1, MEL-18, mph1 and Bmi1 [33,34], suggesting that E2F6 may modulate cell signaling pathways through recruitment of the polycomb transcription factors [9]. Polycomb group complex proteins regulate different cellular functions with PRC1 and PRC2 complex. PRC1 complex is recruited to chromatin through the recognition of H3K27me3 by chromobox (CBX) proteins [35,36], and PRC2 complex is recruited to chromatin which allows tri-methylation of histone H3 Lys27 by EZH2 [35]. RNF2/RING1 homologs that are included in PRC1 complex can catalyze H2A monoubiquitylation at Lys119 [37]. Then RYBP repressor protein recognizes H2A monoubiquitylation and represses transcription activity [38]. Based on these studies, it is also important to suggest the EBNA3C may affect the functions of these cellular antigens and related pathways through E2F6, which may provide new insights for targeting E2F transcription factors in EBV-associated cancers as potential therapeutic intervention strategies.

In regard to EBV-mediated lymphomagenesis, many signaling pathways have been regulated by latent antigens during EBV latent infection [39]. EBV latent antigen EBNA3C, one of the essential proteins for in vitro primary B-cell transformation, was identified as a transcriptional modulator that can efficiently regulate the transcription of both cellular and viral genes [40,41]. It was reported that EBNA3C associates with RBP-Jκ and regulates Notch-induced transcription which was very important for LCL propagation [1]. Additionally, EBNA3C was also shown to interact with a range of transcription factors or regulators, including c-Myc [42], SUMO1, SUMO3 [43], HDAC1 [44], CtBP [45], DP103 [46], p300, Prothymosin-α [47], Nm23-H1 [48], p53 [4], Mdm2 [49], ING4 and ING5 [50]. As a master transcriptional co-regulator, many canonical domains of EBNA3C are essential for protein-protein interactions. Thus, it is possible for EBNA3C to bind with several factors at once or temporally to regulate important cellular pathways including the cell cycle. These functional domains will help us understand how EBNA3C regulates numerous cellular pathways by interacting with multiple proteins. A comprehensive virus-host interaction network implies a complicated regulatory mechanism for EBV-mediated tumorigenesis. Besides, the identification of EBV super-enhancer provides further evidence for the interaction network [51]; suggesting that EBV latent antigens could interact with many proteins at once and regulate special gene-regulatory sites in order to govern cellular functions. The ongoing deep-sequencing data analysis will provide further information to deconstruct the complex virus-host interaction network important for these cellular pathways including gene regulation.

Overall, our work has demonstrated that E2F6 has adopted intricate strategies to target E2F1 for its transcriptional repression and that leads to deregulation of its cellular activities in EBV-mediated pathogenesis. Our study now show a role for EBNA3C in regulating E2F6 expression as well as E2F6 mediated modulation of the activities of E2F1 transcription factor. Furthermore, these results demonstrate how EBNA3C-mediated deregulation of E2F6 alters the cellular proliferative function of B-cells to be transformed into malignant cells. Our current study strongly demonstrates that the EBNA3C-E2F6 interaction promotes cellular proliferation by regulating E2F1 expression. This also offers a novel therapeutic target in E2F6 for targeted killing of EBV-associated B-cell lymphomas.

## Materials and Methods

### Ethics statement

PBMC (human peripheral blood mononuclear cells) were obtained from UPENN immunology core donated by different de-identified healthy donors [52,53]. All the protocols were approved by the Institutional Review Board (IRB) and conducted according to the Declaration of Helsinki. And each donor gave written, informed consent.

### Plasmids, cell lines and antibodies

Myc-tagged EBNA3C full length and truncated mutants, and GFP-tagged EBNA3C expression plasmids were described previously [5]. Flag-tagged and GST-tagged human E2F6 expression construct were generated by inserting human E2F6 gene in pA3F and pGEX2T vector with EcoR1 and Not1 restriction digestion respectively. Wild-type Flag-tagged-E2F1 was described earlier [6], full-length pGL2-basic wild-type human E2F1 promoter construct was kindly provided by Dr. Joseph R. Nevins (Duke University) [54]. The plasmids expressing E2F2, E2F3a, E2F4 and E2F6 were generously provided by Andrew D. Wells (University of Pennsylvania, Philadelphia, PA) and used to generate Flag-tagged expression constructs. Wild-type BAC-GFP-EBV and EBNA3C null mutant (ΔEBNA3C BAC-GFP-EBV) were described previously [52,55].

Mouse anti-Flag (M2) antibody was purchased from Sigma-Aldrich (St. Louis, MO). Hybridomas for mouse anti-Myc (9E10), and anti-EBNA3C (A10) were previously described [6,56]. Mouse anti-E2F6 (TFE61) antibody was purchased from Abcam (Cambridge, MA). Rabbit anti-E2F1 (C-20) antibody was obtained from Santa Cruz Biotechnology (Santa Cruz, CA). Mouse anti-GAPDH antibody was bought from US-Biological (Swampscott, MA).

HEK-293 (human embryonic kidney cell line) and pRb null Saos-2 (human osteosarcoma cell line) were obtained from Jon Aster (Brigham and Woman's Hospital, Boston, MA). U2OS (human osteosarcoma cell line) was purchased from the American Type Culture Collection (ATCC). MEF (mouse embryonic fibroblast cell line) was a gift from Xiaolu Yang (University of Pennsylvania). HEK-293, Saos-2, U2OS and MEF cells were maintained in Dulbecco's modified Eagle's medium (DMEM; Hyclone, Logan, UT) supplemented with 5% fetal bovine serum (FBS), 25 U/ml penicillin, 50μg/ml streptomycin, and 2mM L-glutamine.

EBV-negative Burkitt’s lymphoma cells Ramos and BJAB were kindly provided by Elliot Kieff (Harvard Medical School, Boston, MA). Mutu I and Mutu III cells were kindly provided by Yan Yuan (School of Dental Medicine, University of Pennsylvania, Philadelphia, PA). BJAB stably expressing EBNA3C cells (BJAB7, BJAB10) were prepared by transfecting pZipneo EBNA3C into BJAB cells followed with neomycin selection [57]. LCL1 and LCL2 cells were EBV-transformed immortalized LCLs (lymphoblastoid cell line) generated in our laboratory [58]. Lentivirus-mediated stable EBNA3C knockdown (sh-EBNA3C) or scramble control (sh-ctrl) LCL1 cells generated in our laboratory were previously described [53,59]. PBMC, Ramos, BJAB, BJAB7, BJAB10, LCL1 and LCL2 were grown in RPMI 1640 media (Hyclone, Logan, UT) supplemented as described above.

### Transfections

Bio-Rad Gene Pulser II electroporator was used to transfect HEK-293, Saos-2, MEF and B-cells by electroporation. The electroporation condition is 210 V and 975 μF for HEK-293, Saos-2, MEF cells, and 220 V and 975 μF for Ramos cells. For immunofluorescence assay, cells were transfected with jetPRIME (Polyplus Transfection, Illkirch, France) according to the standard protocols.

### Infection of PBMCs with BAC-GFP-EBV

As previously described [52], 10 million Peripheral blood mononuclear cells (PBMC) were incubated with BAC-GFP-EBV supernatant, with a multiplicity of infection (MOI) of 5, in 1ml of RPMI 1640 media with 10% FBS. Cells were collected after 4 hours incubation at 37°C, re-suspended in 2ml of complete RPMI 1640 media, and cultured in 6-well plate. EBV GFP expression was measured by fluorescence microscopy. After indicated times of post-infection, the cells were harvested and detected expression levels. The results were derived from three independent experiments.

### Real-time quantity PCR

Quantitative real-time PCR (qPCR) analysis was carried out as previously described [49]. Briefly, total RNA was isolated using Trizol reagent (Invitrogen, Inc., Carlsbad, CA), treated with Dnase I (Invitrogen), and reversed to cDNA by using High-Capacity cDNA Reverse Transcription Kit (Applied Biosystems Inc., Foster City, CA), according to the manufacturer’s instructions. Real-time PCR was performed using a Power SYBR Master Mix kit (Applied Biosystems) and the Step One Plus Real-time PCR system (Applied Biosystems). The results were normalized to the endogenous control, GAPDH. Each sample was performed in triplicate. The primers used in this study are listed in S1 Table.

### GST pull-down assay

The constructs of full length EBNA3C and its different truncated mutants were used to produce *in vitro* translated proteins using the T7-TNT transcription/translation kit (Promega). Cell lysates from different B-cells (BJAB, BJAB7, BJAB10, LCL1, LCL2) were harvested and prepared. The translated proteins or cell lysates were co-incubated with GST control protein or purified GST-E2F6 protein from bacteria, followed by addition of GST beads. Then the protein mixtures were washed three times with binding buffer (0.1% NP-40, 0.5 mM DTT, 10% glycerol, and protease inhibitors in 1X PBS), analyzed by 10% SDS-PAGE, and detected with western blot using mouse anti-EBNA3C antibody (A10).

### Co-immunoprecipitation and western blot analysis

Cells were washed with 1X Phosphate Buffered Saline (PBS) for twice, and lysed in RIPA buffer (10 mM Tris pH 7.5, 0.5% NP-40, 2 mM EDTA, 150 mM NaCl) with 1 mM PMSF and protease inhibitors for 1 hour at 4°C. After pre-cleaning with normal mouse/rabbit serum and 35 μl protein-A/G (1:1 mixture)-coupled Sepharose beads, cell lysates were incubated with appropriate antibody (1 μg/ml) to capture the interest proteins by rotating overnight at 4°C. Immunoprecipitations were washed three times in RIPA buffer. To perform western blot analysis, the immunoprecipitated samples and 5% of total cell lysates were separated by SDS-PAGE and transferred to nitrocellulose membranes, followed by incubating with appropriate antibodies and scanning using the Odyssey scanner (LiCor Inc., Lincoln, NE).

### Immunofluorescence analysis

U2OS or Saos-2 cells were transfected with different expression constructs using jetPRIME (Polyplus Transfection, Illkirch, France). After 36 hours post-transfection, cells were fixed and permeabilized with 4% paraformaldehyde (PFA) and 0.1% Triton X-100 followed by blocking with 5% BSA at room temperature. Flag-tagged E2F6 was incubated with mouse anti-Flag (M2) antibody and Alexa Fluor 594-conjugated secondary antibody (Molecular Probes, Invitrogen, Carlsbad, CA), while GFP-tagged EBNA3C was detected by GFP-fluorescence directly. BJAB, BJAB10, and LCL1 cells were semi-air-dried on slides in culture hood and fixed as mentioned earlier. Endogenous EBNA3C and E2F6 proteins were detected with their specific primary antibodies and the corresponding secondary antibodies. Nucleus were stained with DAPI (4′,6′,-diamidino-2-phenylindole; Pierce, Rockford, IL). Finally, the cells on slides were washed in 1X PBS for three times and mounted in an antifade mounting media. The images were collected by Fluoview FV300 confocal microscope and FLUOVIEW software (Olympus Inc., Melville, NY) was used for image analysis.

### Stability assay

10 million MEF or Saos-2 cells were transfected with combinations of constructs by using the BioRad electroporation system. After 36 hours post-transfection, the transfected cells as well as indicated B-cells were incubated with 40 μg/ml cycloheximide (CHX) at specific time points. Then cells were harvested, lysed with RIPA buffer, and analyzed by western blot. Odyssey 3.0 software was used to quantify the band intensities.

### Luciferase assay

Luciferase assays were performed as previously described with few modifications [27]. Briefly, 10 million HEK-293 or Saos-2 cells were transfected with luciferase reporter and different combination of plasmids as indicated. At 36 hours post-transfection, cells were harvested and lysed for luciferase assay using dual luciferase assay system (Promega). The luciferase as well as renilla activities were measured using LMaxII384 luminometer (Molecular Devices, Sunnyvale, CA). The results represent experiments performed in duplicate.

### Chromatin immunoprecipitation assay

Chromatin immunoprecipitation (ChIP) was performed as previously described [60]. Briefly, 30 million HEK-293 cells were transiently transfected by electroporation with E2F1 luciferase reporter plasmid or control vector, and expression constructs Myc-EBNA3C or Flag-E2F6. 48h of post-transfection, cells were cross-linked by 1% formaldehyde, collected, sheared DNA to an average length of 600bp by sonication, as confirmed by agarose gel electrophoresis. Cross-linked DNA was immunoprecipitated with anti-E2F6 antibody or normal IgG and subjected for Real-time PCR analysis with primers designed for specific regions of E2F1 promoter. The primers used in this assay are: for exogenous E2F1 promoter 5'-GGTACCATCCGGACAAAG-3' and 5'-GGTTCCTATTGGCTTTAACG-3'; for endogenous E2F1 promoter 5'-GCAGCAGTGGGCAATAGA-3' and 5'-CACCGGAATCCCTGTAAT-3' [10]. For B cell lines, about 50 million cells were harvested, immunoprecipitated with either E2F6 specific antibody or normal IgG and processed as above. The experiments were performed in duplicate and standard deviations (SDs) were indicated by error bars.

### Lentiviral production and infection

The two sense strands of E2F6 shRNA are 5’-tcgagtgctgttgacagtgagcgaAAGGATTGTGCTCAGCAGCTGtagtgaagccacagatgtaCAGCTGCTGAGCACAATCCTTgtgcctactgcctcggaa–3’ (sh-E2F6-1), and 5’- tcgagtgctgttgacagtgagcgaTTGATGTATCGCTGGTTTATTtagtgaagccacagatgtaAATAAACCAGCGATACATCAAgtgcctactgcctcggaa–3’ (sh-E2F6-2), respectively. The upper-cases designate E2F6 target sequences, while lower cases specify hairpin and sequences. These sense stranded oligos were annealed with their respective anti-sense stranded oligos, and then cloned into pGIPZ vector using Xho I and Mlu I restriction sites. Besides, a sh-ctrl plasmid including control shRNA sequence 5’-TCTCGCTTGGGCGAGAGTAAG–3’ (Dharmacon Research, Chicago, IL) was used as a negative control. Lentivirus production and transduction of B-cell lines has been described previously [53].

### CSFE proliferation assay

B-cells were collected and suspended in 1 X PBS at a concentration of 10 X 10^6^ cells/ml. Then CFSE solution was added to make a final concentration of 5μM. Equal volume of 1X PBS containing 5% FBS was added after 10 mins incubation at room temperature. Cells were washed three times with 1X PBS containing 5% FBS and equally divided into several plates for incubation. At different time points (0, 24h, 48h, 72h), cells were harvested, washed and fixed in 4% PFA. The cells were washed 3 more times with ice-cold 1 X PBS and resuspended in 5000μl 1 X PBS, then analyzed on FACScalibur cytometer (Becton-Dickinson Inc., San Jose, CA) and FlowJo software (Treestar, Inc., San Carlos, CA).

### Statistical analysis

Data represented are as the mean values with standard errors of means (SEM) or standard deviation (SD). Statistical significance of differences in the mean values was analyzed using the 2-tailed student's t-test. P-value below 0.05 was considered here as significant (*P < 0.05; **P < 0.01; NS, not significant).

### Accession numbers

Epstein-Barr virus (EBV) genome, strain B95-8-GenBank: V01555.2, EBNA3C (Human herpesvirus 4)-NCBI Reference Sequence: YP_401671.1, E2F1 (Homo sapiens)-NCBI Reference Sequence: NM_005225.2, E2F2 (Homo sapiens)-NCBI Reference Sequence: NM_004091.3, E2F3 (Homo sapiens)-NCBI Reference Sequence: NM_001949.4, E2F4 (Homo sapiens)-NCBI Reference Sequence: NM_001950.3, E2F6 (Homo sapiens)-NCBI Reference Sequence: NM_198256.3.

## Supporting Information

S1 Fig EBNA3C interacts with E2F6 and increases its stability. A) HEK-293 cells were transfected with Myc-tagged EBNA3C or Flag-tagged E2F6, collected after 48 hours transfection, immune-precipitated with rabbit anti-E2F6 antibody, and detected with western blot. B) 10 million Mutu I or Mutu III were lysed and analyzed with western blot. C) HEK-293 cells were transfected with Flag-tagged E2F6 and control vector or Myc-tagged EBNA3C. At 24 hours post-transfection, transfected cells were incubated with cycloheximide (CHX) for indicated times, then collected and analyzed with western blot.
(TIF)

S2 Fig EBNA3C expression is necessary for E2F6 stability after EBV infection. A) BJAB cells were infected with wild-type EBV-GFP-BAC or ΔEBNA3C BAC-GFP-EBV, respectively. Cells were collected after indicated times and extracted total RNA with Trizol according to the manufacturer’s instructions. The levels of E2F6 were quantified using Real-time PCR with GAPDH as an internal control. *P < 0.05; **P < 0.01; NS, not significant, compared with the control group. B) BJAB infected with wild-type EBV-GFP-BAC were harvested and isolated total RNA at indicated times. The levels of EBNA3C were detected with Real-time PCR.
(TIF)

S3 Fig Inhibition of EBNA3C-related E2F1 promoter activity is mediated by E2F6. HEK-293 cells were co-transfected with control vector, sh-Ctrl, or sh-E2F6-1 plasmid in the presence of control vector or EBNA3C. After 48 hours post-transfection, cells were collected and lysed, then E2F1 promoter activity was detected.
(TIF)

S4 Fig EBNA3C mutants containing E2F6-binding domain are co-localized with E2F6 in human cells. Indicated Myc-tagged EBNA3C mutants were transfected into HEK-293 cells with Flag-tagged E2F6. The cells were then fixed, incubated with appropriated primary and secondary antibodies at 48 hours post-transfection, and visualized using confocal microscopy.
(TIF)

S5 Fig E2F6 knock-down affects EBNA3C-mediated cell proliferation in LCL1 cells. A) Selected E2F6 knock-down stable LCL1 cells with GFP fluorescence were checked with fluorescence microscope. B) 10^5^ E2F6 knock-down (sh-E2F6-1) or control (sh-Ctrl) LCL1 cell lines were cultured in 6-well plate. Then cell numbers were counted at indicated time using trypan blue dye exclusion technique.
(TIF)

S6 Fig The interaction network among E2F family members. Using Ingenuity Pathway Analysis (IPA), the interactions among E2F family members were generated from the common database of molecular interactions in the program.
(TIF)

S1 Table Summary of primers used for Real-time PCR in this study. (DOCX)
