# Supplementary figures and images for: EBV Nuclear Antigen 3C Mediates Regulation of E2F6 to Inhibit E2F1 Transcription and Promote Cell Proliferation
Source: PLoS Pathog. 2016 Aug 22;12(8):e1005844. doi: 10.1371/journal.ppat.1005844 (PMC4993364; doi:10.1371/journal.ppat.1005844)

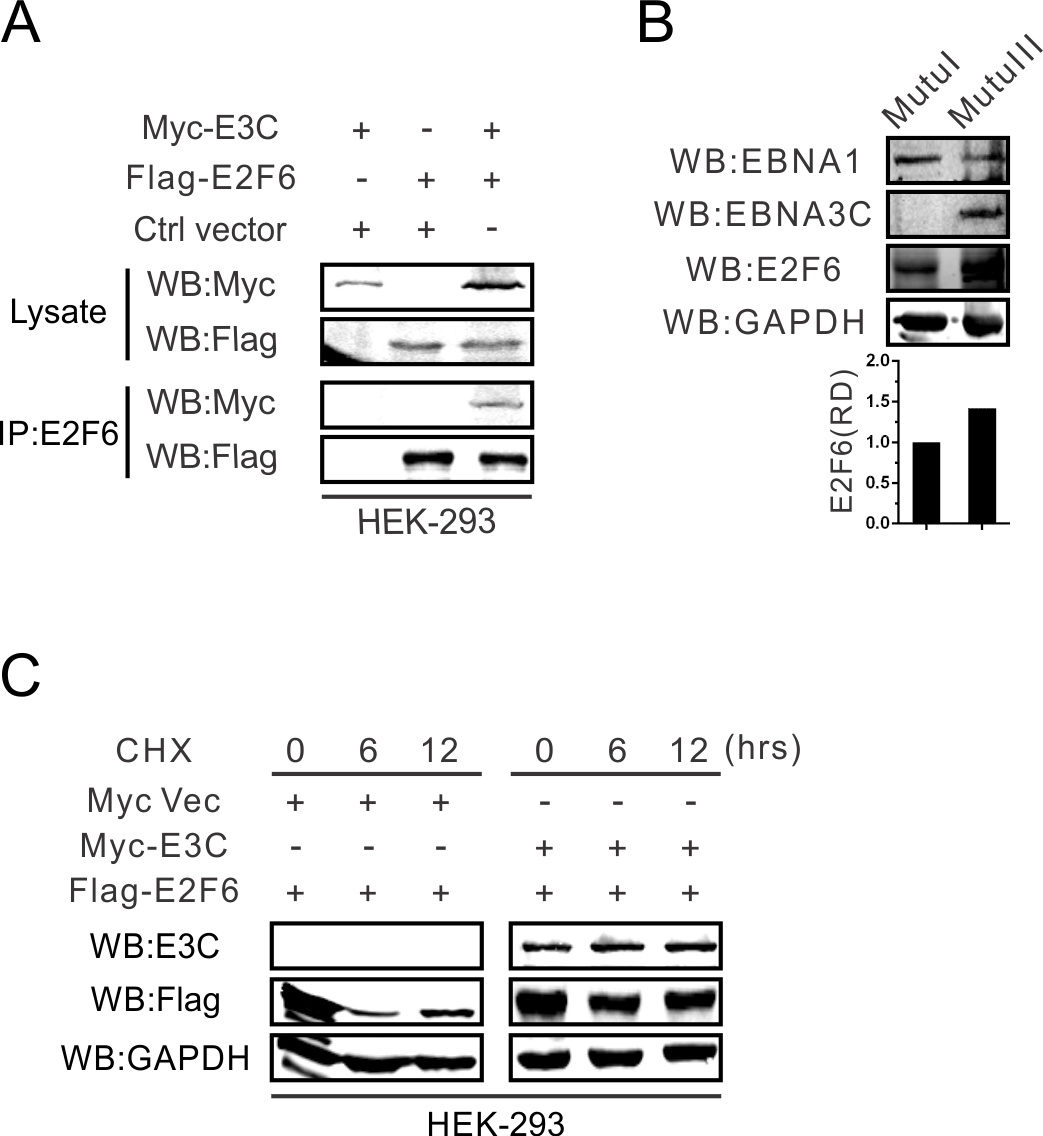

Supplement: S1 Fig — A) HEK-293 cells were transfected with Myc-tagged EBNA3C or Flag-tagged E2F6, collected after 48 hours transfection, immune-precipitated with rabbit anti-E2F6 antibody, and detected with western blot. B) 10 million Mutu I or Mutu III were lysed and analyzed with western blot. C) HEK-293 cells were transfected with Flag-tagged E2F6 and control vector or Myc-tagged EBNA3C. At 24 hours post-transfection, transfected cells were incubated with cycloheximide (CHX) for indicated times, then collected and analyzed with western blot. (TIF) [file ppat.1005844.s001.tif]

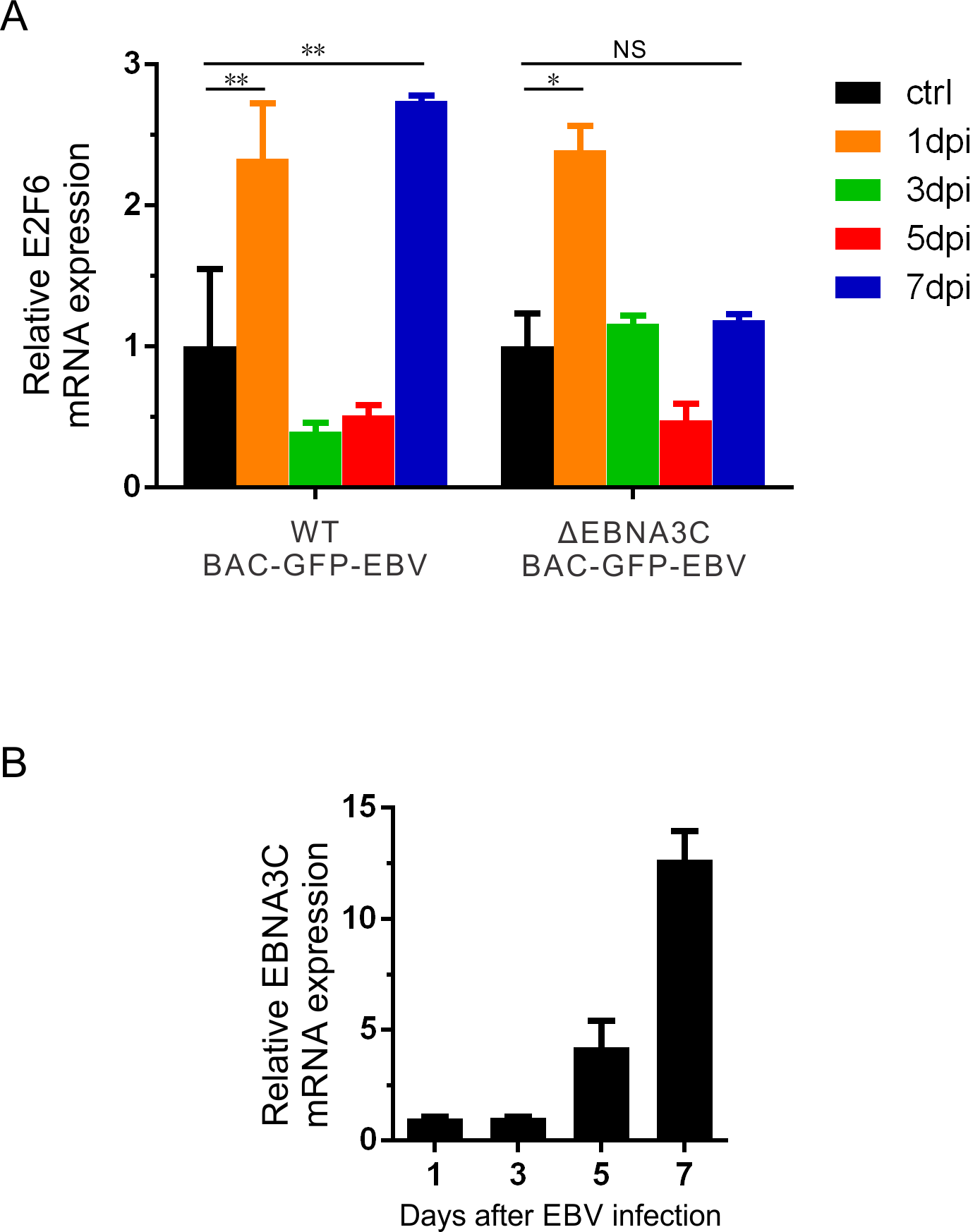

Supplement: S2 Fig — A) BJAB cells were infected with wild-type EBV-GFP-BAC or ΔEBNA3C BAC-GFP-EBV, respectively. Cells were collected after indicated times and extracted total RNA with Trizol according to the manufacturer’s instructions. The levels of E2F6 were quantified using Real-time PCR with GAPDH as an internal control. *P < 0.05; **P < 0.01; NS, not significant, compared with the control group. B) BJAB infected with wild-type EBV-GFP-BAC were harvested and isolated total RNA at indicated times. The levels of EBNA3C were detected with Real-time PCR. (TIF) [file ppat.1005844.s002.tif]

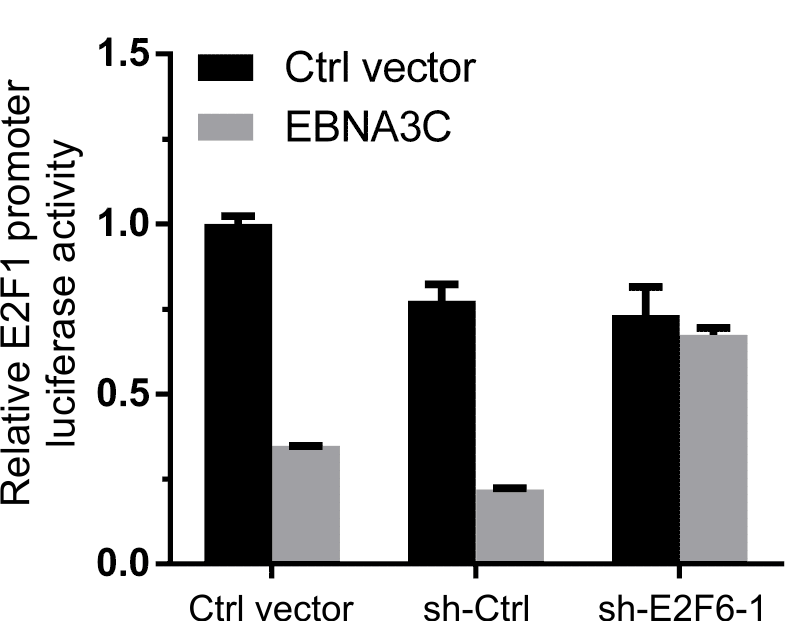

Supplement: S3 Fig — HEK-293 cells were co-transfected with control vector, sh-Ctrl, or sh-E2F6-1 plasmid in the presence of control vector or EBNA3C. After 48 hours post-transfection, cells were collected and lysed, then E2F1 promoter activity was detected. (TIF) [file ppat.1005844.s003.tif]

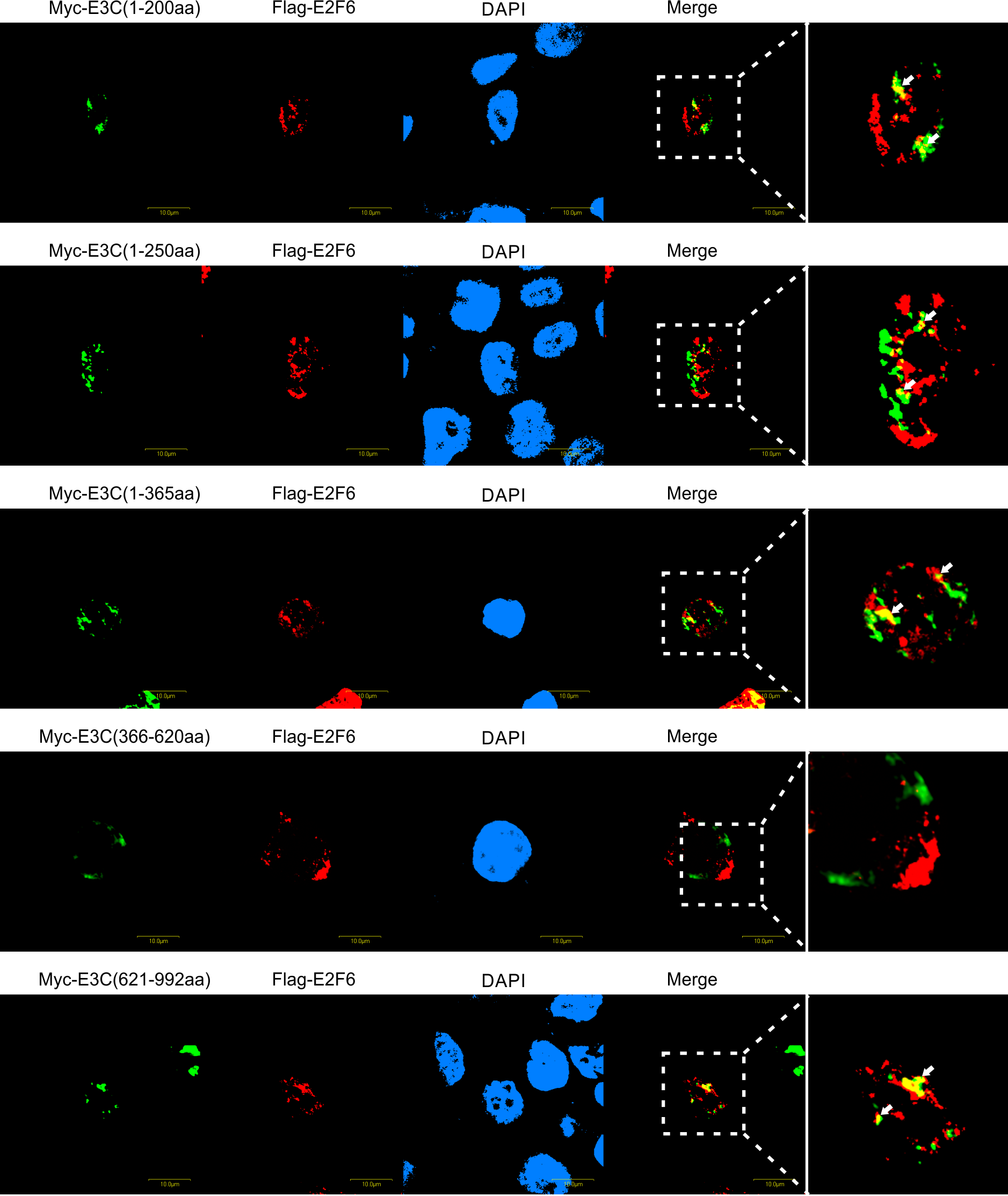

Supplement: S4 Fig — Indicated Myc-tagged EBNA3C mutants were transfected into HEK-293 cells with Flag-tagged E2F6. The cells were then fixed, incubated with appropriated primary and secondary antibodies at 48 hours post-transfection, and visualized using confocal microscopy. (TIF) [file ppat.1005844.s004.tif]

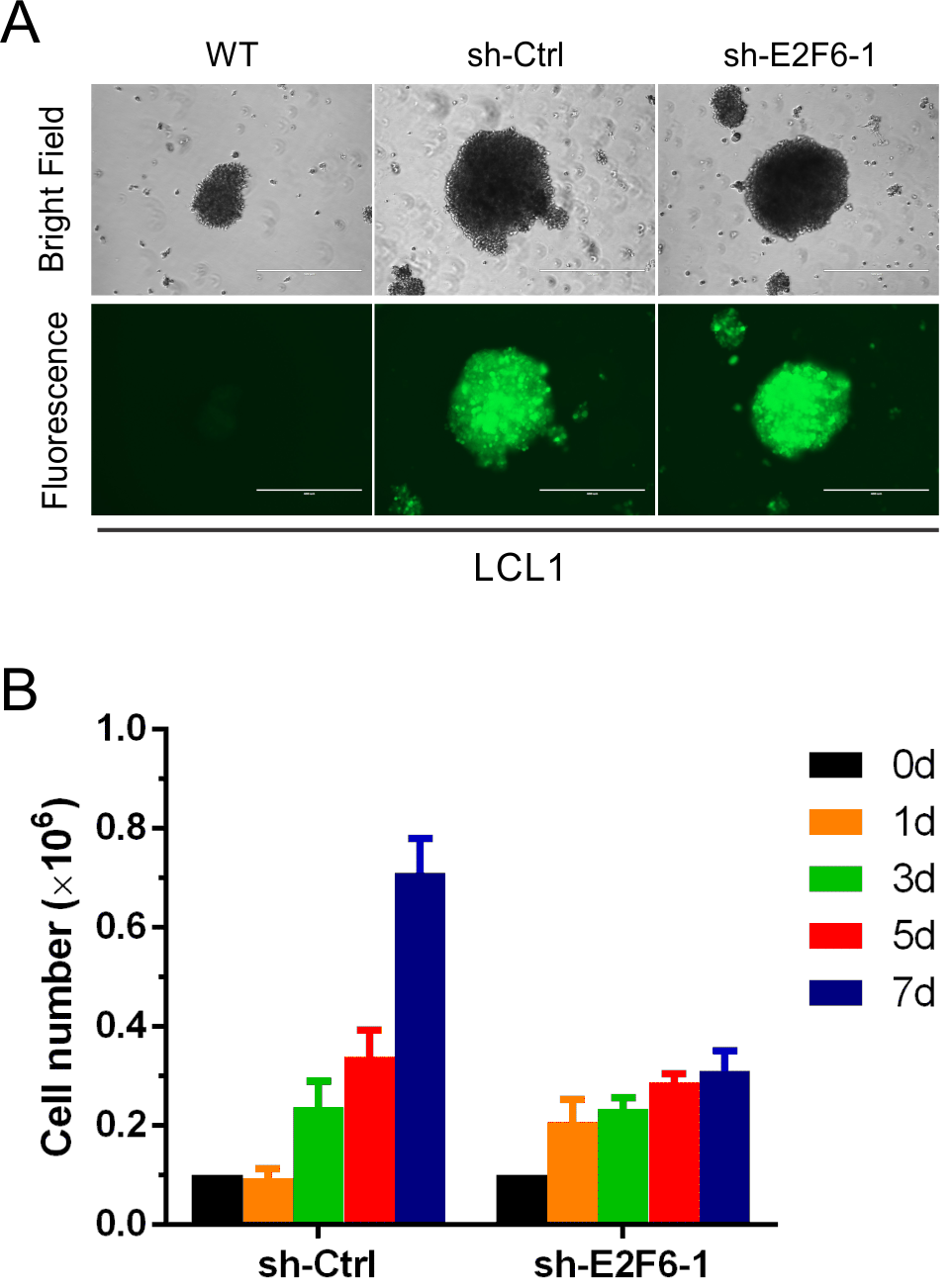

Supplement: S5 Fig — A) Selected E2F6 knock-down stable LCL1 cells with GFP fluorescence were checked with fluorescence microscope. B) 105 E2F6 knock-down (sh-E2F6-1) or control (sh-Ctrl) LCL1 cell lines were cultured in 6-well plate. Then cell numbers were counted at indicated time using trypan blue dye exclusion technique. (TIF) [file ppat.1005844.s005.tif]

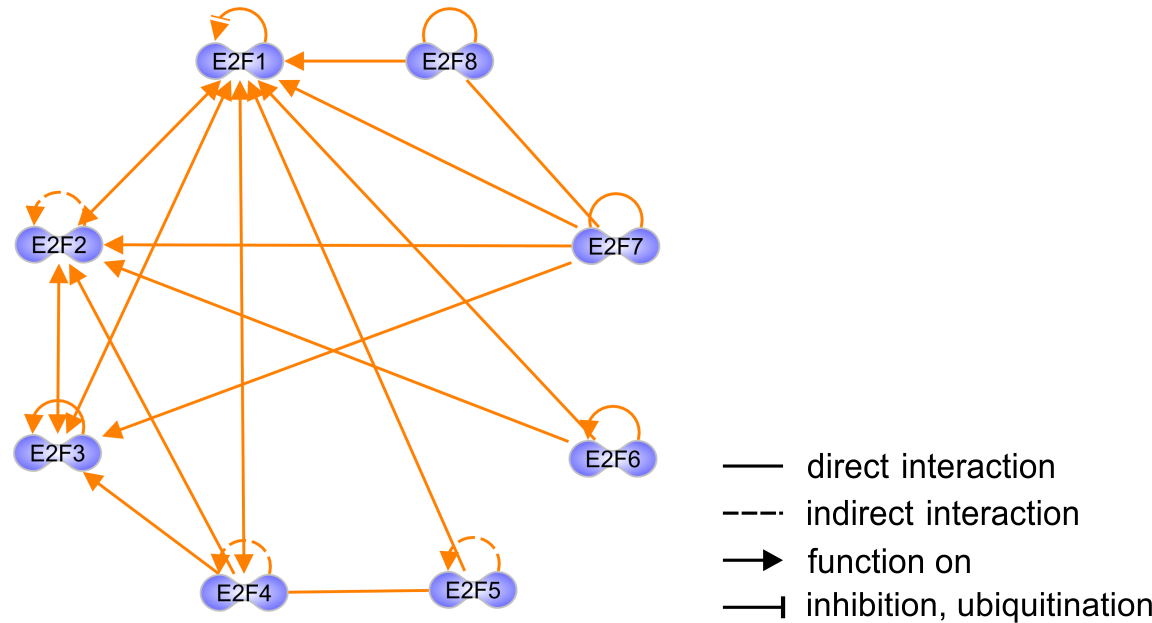

Supplement: S6 Fig — Using Ingenuity Pathway Analysis (IPA), the interactions among E2F family members were generated from the common database of molecular interactions in the program. (TIF) [file ppat.1005844.s006.tif]
