# Supplementary material for: EBV Nuclear Antigen 3C Mediates Regulation of E2F6 to Inhibit E2F1 Transcription and Promote Cell Proliferation
Source: PLoS Pathog. 2016 Aug 22;12(8):e1005844. doi: 10.1371/journal.ppat.1005844 (PMC4993364; doi:10.1371/journal.ppat.1005844)
Supplement: S1 Table — (DOCX) [file ppat.1005844.s007.docx]

**Table 1. Summary of primers used for Real-time RT-PCR in this study.**

| **Gene** | **Forward sequence** | **Reverse sequence** | **Size, bp** |
| --- | --- | --- | --- |
| E2F1 | 5’-GGCCAGGTACTGATGGTCA-3’ | 5’-GACCCTGACCTGCTGCTCT-3’ | 129 |
| E2F2 | 5’-TTGGGAACTCAGGGACGA-3’ | 5’-CAAGTTGTGCGATGCCTG-3’ | 94 |
| E2F3 | 5’-CTAGCTCCAGCCTTCGCTTT-3’ | 5’-AGCCTCCTCTACACCACGC-3’ | 127 |
| E2F4 | 5’-CACTCTCGTGTGGGATCAAA-3’ | 5’-GGACCCAACCCTTCTACCTC-3’ | 104 |
| E2F5 | 5’-TTTTGCCTCACAGCCAAAGT-3’ | 5’-CACTACCAAGTTCGTGTCGC-3’ | 90 |
| E2F6 | 5’-AGAGCTTTTCTCATGGACACA-3’ | 5’-CGAGACCCCATCAACGTG-3’ | 92 |
| EBNA3C | 5′-AGAAGGGGAGCGTGTGTTGT-3′ | 5′-GGCTCGTTTTTGACGTCGGC-3′ | 153 |
| GAPDH | 5′-TGCACCACCAACTGCTTAG-3′ | 5′-GATGCAGGGATGATGTTC-3′ | 176 |
